# In Situ Metabolic Characterisation of Breast Cancer and Its Potential Impact on Therapy

**DOI:** 10.3390/cancers12092492

**Published:** 2020-09-03

**Authors:** Gábor Petővári, Titanilla Dankó, Anna-Mária Tőkés, Enikő Vetlényi, Ildikó Krencz, Regina Raffay, Melinda Hajdu, Dániel Sztankovics, Krisztina Németh, Krisztina Vellai-Takács, András Jeney, Janina Kulka, Anna Sebestyén

**Affiliations:** 1 1st Department of Pathology and Experimental Cancer Research, Semmelweis University, Üllői út 26, H-1085 Budapest, Hungary; gaborpetovari@gmail.com (G.P.); tita.danko@gmail.com (T.D.); eniko.vetlenyi@gmail.com (E.V.); krencz.ildiko@gmail.com (I.K.); regiraffay@gmail.com (R.R.); melindahajdu@gmail.com (M.H.); sztankovics.daniel@gmail.com (D.S.); ajeney7@gmail.com (A.J.); 2 2nd Department of Pathology, Semmelweis University, Üllői út 93, H-1091 Budapest, Hungary; tokesa1972@yahoo.co.uk (A.-M.T.); janinakulka@gmail.com (J.K.); 3 MS Metabolomics Laboratory, Core Facility, Research Centre for Natural Sciences, Magyar Tudósok Blvd 2, H-1117 Budapest, Hungary; nemeth.krisztina.94@ttk.hu; 4 Department of Biological Anthropology, Eötvös Loránd University, Pázmány Péter sétány 1/A, H-1117 Budapest, Hungary; takacsve@gmail.com

**Keywords:** mTOR activity, metabolic characterisation, metabolic plasticity, breast cancer, metabolism

## Abstract

**Simple Summary:**

Metabolic rewiring, as an important hallmark of cancer supports the bioenergetic needs of growing tumours at the primary site or in metastases. Mammalian target of rapamycin (mTOR) hyperactivity may contribute to metabolic plasticity and progression in cancers. We set out to assess the metabolic complexity in breast cancer cell lines and primary breast cancer cases. Using a set of immunoreactions, we observed that the characterisation of metabolic pathways has a prognostic potential in human cases. High in situ metabolic plasticity was considered with high mTOR activity and high expression of at least two other studied metabolic enzymes (lactate dehydrogenase A, glutaminase, fatty acid synthase or carnitine palmitoyltransferase). According to our metabolic analyses and immunohistochemistry results, breast cancer is characterised by considerable metabolic diversity in individual cases. Consequently, we suggest selecting patients who may benefit from more accurate follow-up and specific therapies (including the combination of anti-metabolic treatments and recent therapies).

**Abstract:**

In spite of tremendous developments in breast cancer treatment, the relatively high incidence of relapsing cases indicates a great need to find new therapeutic strategies in recurrent, metastatic and advanced cases. The bioenergetic needs of growing tumours at the primary site or in metastases—accumulating genomic alterations and further heterogeneity—are supported by metabolic rewiring, an important hallmark of cancer. Adaptation mechanisms as well as altered anabolic and catabolic processes balance according to available nutrients, energy, oxygen demand and overgrowth or therapeutic resistance. Mammalian target of rapamycin (mTOR) hyperactivity may contribute to this metabolic plasticity and progression in breast carcinomas. We set out to assess the metabolic complexity in breast cancer cell lines and primary breast cancer cases. Cellular metabolism and mTOR-related protein expression were characterised in ten cell lines, along with their sensitivity to specific mTOR and other metabolic inhibitors. Selected immunohistochemical reactions were performed on ~100 surgically removed breast cancer specimens. The obtained protein expression scores were correlated with survival and other clinicopathological data. Metabolic and mTOR inhibitor mono-treatments had moderate antiproliferative effects in the studied cell lines in a subtype-independent manner, revealing their high adaptive capacity and survival/growth potential. Immunohistochemical analysis of p-S6, Rictor, lactate dehydrogenase A, glutaminase, fatty acid synthase and carnitine palmitoyltransferase 1A in human samples identified high mTOR activity and potential metabolic plasticity as negative prognostic factors for breast cancer patients, even in subtypes generally considered as low-risk. According to our results, breast cancer is characterised by considerable metabolic diversity, which can be targeted by combining antimetabolic treatments and recent therapies. Alterations in these pathways may provide novel targets for future drug development in breast cancer. We also propose a set of immunostainings for scoring metabolic heterogeneity in individual cases in order to select patients who may benefit from more accurate follow-up and specific therapies.

## 1. Introduction

The number of newly diagnosed breast cancer patients is increasing, and it may reach 2.5–3 million cases worldwide per year over the next decades, despite recent developments in prevention, screening and therapy [1]. Considerable efforts have been made by NGS sequencing and other “omic” studies to improve the molecular characterisation of the major subtypes—luminal A (LumA), luminal B (LumB1 and B2), HER2 enriched (HER2+) and basal-like (the large majority of triple-negative breast cancer—TNBC)—and to find novel targets for personalised therapy. Several signalling pathways and crossroads have been selected as potential targets: hormone receptors (HRs), growth factor receptors (HER2, EGFR, VEGFR), phosphoinositide-3-kinase (PI3K), Akt and mTOR as well as targets in regulatory functions such as the cell cycle, programmed cell death or immunoediting (e.g., CDK4/6, PARP and PD-1/PD-L1, respectively) subtype dependently. Despite new treatment options, the incidence of relapse and therapy resistance in advanced disease indicate a great need for new treatment strategies. Finding new biomarkers and establishing more appropriate patient eligibility criteria are also necessary [1,2].

One of the main features leading to therapy resistance is tumour heterogeneity. Tumour cells are surrounded by other cells and matrix elements in their micro- and macroenvironment. In this heterogeneous tissue, malignant cells—depending on their genetic instability—accumulate several genomic alterations, which generate further diversity. Moreover, the selective pressure of tumour ecosystems can lead to tumour evolution and divergent clonal selection at the primary site or in metastases [3,4,5].

The special cellular energetic needs of tumour growth and survival are connected with metabolic alterations. Several studies on tumour metabolism have suggested that metabolic rewiring may be an important hallmark of cancer. Metabolic adaptation mechanisms could alter anabolic and catabolic processes’ (such as glycolysis, oxidative phosphorylation—OXPHOS, glutaminolysis, lipid oxidation and lipid/nucleotide/protein synthesis) balance according to available nutrients, energy and oxygen and the demands of growing cell numbers in a highly diverse microenvironment influenced by overgrowth and/or therapeutic pressure [6]. Since the discovery of rapamycin (RAPA) and its target, mTOR, numerous studies have concluded that mTOR signalling is a “meeting point” in metabolic reprogramming, coordinating extra- and intracellular energy sources for metabolic intermediate and macromolecule syntheses. As a component of two distinct protein complexes (mTORC1 and C2), mTOR kinase is involved in multiple functions regulating cell growth, proliferation and survival, through cellular metabolic alterations such as protein synthesis, glucose, glutamine, lipid metabolism, pentose phosphate and one-carbon metabolism-related epigenetic pathways. Hyperactive mTOR signalling is known to occur in different cancers, including breast carcinomas, and additionally, it may contribute to metabolic plasticity and tumour progression [7,8].

**Figure 1:**
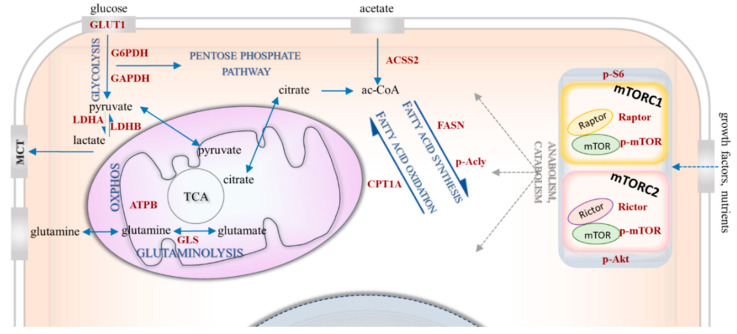
Bioenergetic mechanisms in metabolic rewiring. The simplified figure shows the investigated bioenergetic pathways with the metabolic enzymes and proteins of interest. The mTOR-related proteins include p-S6, p-(Ser473)-Akt (p-Akt), p-mTOR, Raptor, Rictor. The metabolic enzymes/proteins include GLUT1 (glucose transporter 1), GAPDH (glyceraldehyde 3-phosphate dehydrogenase), G6PDH (glucose-6-phosphate dehydrogenase), LDHA (lactate dehydrogenase A) and LDHB (lactate dehydrogenase B) involved in glycolysis; GLS (glutaminase) involved in glutaminolysis; ACSS2 (acyl-coA synthetase short-chain family member 2) involved in acetate consumption; and FASN (fatty acid synthase), p-Acly (phospho-ATP-citrate lyase) and CPT1A (carnitine palmitoyltransferase 1A) involved lipid metabolism.

Targeting metabolic rewiring—influenced by several bioenergetic mechanisms (Figure 1)—is a promising treatment option for various malignancies. Of note, the association between breast cancer risk and metabolic syndrome in postmenopausal women [9], as a sign of this bioenergetic adaptation, certainly deserves special attention. Metabolic alterations such as glycolytic hyperactivation, glutamine uptake, lipid metabolism or mTOR activity have been the subject of intensive studies of breast cancer research [10,11,12,13,14]. Several attempts have been made to subclassify these tumours based on their metabolic characteristics [15,16,17]. Despite accumulating data highlighting the correlation between drug resistance and specific metabolic alterations, these studies were primarily focused on cell line-based experiments and/or the relevance of only one metabolic alteration. Some of these studies were completed with expression analysis of molecules related to regulatory failure (e.g., p53 or pyruvate kinase 2—PKM2), enzymes (e.g., PI3K/Akt/mTOR) or transporters (e.g., glucose transporter 1—GLUT1—or monocarboxylate transporters—MCTs) of this network [15,18,19,20]. However, the emerging role of aerobic glycolysis in breast cancer has been reviewed recently; several questions still remain in relation to targeting Warburg-type glycolysis [20]. Glutamine consumption is also a mechanism, which is suggested that could support drug resistance. In addition, a publication highlights the importance of increased glutaminase (GLS) expression in endocrine-resistant breast cancer [21]. Lipid metabolism-related alterations can also contribute to malignant transformation. Moreover, the prevalence of breast cancer has been reported to strongly correlate with obesity [22,23]. The enhanced expression and increased activity of fatty acid synthase (FASN) have been shown to be early events in breast cancer progression [24]. It is reported that cancer cells use fatty acids (FAs) for structural and energy sources as well as signalling purposes. Deregulated lipid metabolism and enhanced FA synthesis have been described in different types of breast cancer: for example, FASN can be upregulated by HER2 signalling and induced by sterol regulatory element-binding protein 1 (SREBP1) [22] under the control of mTOR. Carnitine palmitoyltransferase 1A (CPT1A)—involved in transferring long-chain FAs to the mitochondrial matrix—may have a special role in nutrient deprivation or other stress situations originating from the microenvironmental niche of tumours [23]. Autophagy-dependent cellular degradation processes—as interim survival/adaptation mechanisms—can also have important implications. To orchestrate consumption and nutrient/building block availability, lipogenesis and lipolysis (i.e., anabolism and catabolism, respectively) are being switched on and off, especially in the breast. Breast tissue also contains varying amounts of fat, and its structure can be altered in a hormone-dependent manner. 

The outcome of breast cancer has improved due to novel targeted therapies: between 1985 and 2016, survival time increased by more than 20 months and survival rates by 15% [2]. However, further efforts are necessary to find solutions for patients with poor prognosis. Precision medicine relies on finding key biomarker alterations related to tumour progression; in this context, intra-tumoral heterogeneity is also a critical issue. 

In the present study, the metabolic properties of breast cancer cell lines were characterised by their protein expression profiles and sensitivity to mTOR and metabolic inhibitors. Based on in vitro experiments, immunohistochemistry (IHC) markers were selected and used to evaluate the complexity of protein expression in tumour tissues. To find potentially active or inducible metabolic pathways, which could lead to tumour growth, the stainings were further analysed. As a result, certain stainings were correlated with survival and/or additional clinicopathological data. We show that tumour cell populations can be highly diverse, and metabolic heterogeneity can contribute to worse prognosis in breast cancer.

## 2. Results

### 2.1. mTOR Complex Activity and Inhibitor Sensitivity in Human Breast Cancer Cell Lines

**Figure 2:**
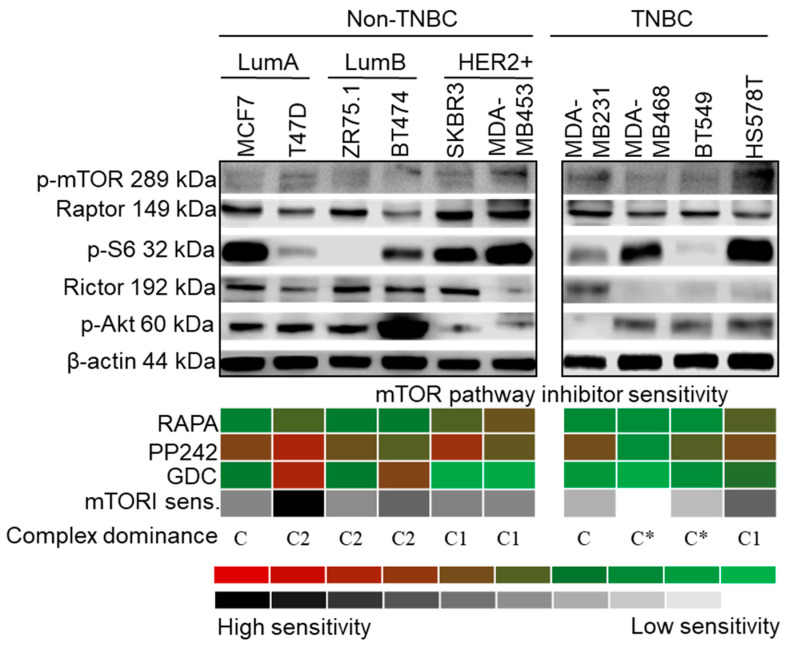
Characteristics of mTOR activity in breast cancer cell lines. Representative Western blots show high mTORC1 and/or C2 activity protein expression patterns (C1—p-mTOR, Raptor and p-S6; C2—p-mTOR, Rictor and p-(Ser473)-Akt (p-Akt)), in a cell line-dependent, subtype-independent manner. In vitro sensitivity to rapamycin (RAPA—50 ng/mL), PP242 (1 µM) and GDC (1 µM) (inhibitors of C1, C1/C2 and Akt, respectively) treatment (72 h) is presented with a colour scheme showing the inhibitory effects, based on the averages of Alamar Blue and sulforhodamine B (SRB) test results: green/white (0%, resistant cells) to red/black (~90–100%, highly sensitive cells). Sensitivity to inhibitors of the mTOR pathway was calculated by adding the intensity scores of the three inhibitors. For determining the distribution of active mTOR complexes—referred as complex dominance—protein expression and inhibitor sensitivity in individual cell lines were taken into account. (C1: C1 complex dominant; C2: C2 complex dominant; C: no complex activity dominance; C*: mTOR inhibitor-resistant phenotype) (loading control: β-actin; triple negative breast cancer—TNBC; luminal A—LumA; luminal B—LumB; HER2+ subtypes).

mTORC1 and C2 complex activity and the antiproliferative effects of in vitro treatments were evaluated in ten human breast cancer cell lines which represent all breast cancer subtypes.

p-mTOR was detected in the examined cell lines, indicating mTOR kinase activity. p-mTOR and Raptor expression did not correlate with the amount of ribosomal p-S6, which is considered to be a useful marker of mTORC1 inhibition sensitivity. The decreasing level of this phospho-protein upon RAPA treatment has been previously shown in mTORC1 inhibitor-sensitive cell lines in a cell type-independent manner. p-S6 was either barely or not detectable in ZR-75.1 and BT549 cells. In parallel, considerable amounts of Rictor and p-(Ser473)-Akt (p-Akt) highlighted potential mTORC2 complex activity in the majority of the studied cells (Figure 2).

RAPA (an mTORC1 inhibitor) slightly inhibited the growth of almost all cell lines in correlation with mTOR activity status, but two cell lines (MDA-MB468 and BT549) seemed to be extremely mTOR and Akt inhibitor (GDC)-resistant. Higher RAPA sensitivity was observed in SKBR3, MDA-MB453, HS578T and T47D cells. GDC exerted a higher antiproliferative effect than RAPA in T47D and BT474 cells. As was expected, the mTORC1/C2 inhibitor PP242 was the most effective inhibitor of PI3K/Akt/mTOR signalling, and it decreased the number of viable cells below 50% in eight cell lines, except for the two resistant cell types (Figure 2, Table S1).

The evaluation of protein expression and inhibitor sensitivity allowed us to establish three categories depending on which mTOR complex showed dominant activity (Figure 2): C2 dominance was determined if all the following three criteria were fulfilled—(a) antiproliferative response to all inhibitors, (b) greater sensitivity to GDC and PP242 than to RAPA, and (c) significantly higher amounts of p-(Ser473)-Akt than p-S6; C1 dominance was assigned to cell lines with lower p-(Ser473)-Akt expression compared to that of p-S6, which demonstrated a lower response to GDC treatment; no complex dominance or balanced mTORC1/C2 activity was defined in cases where RAPA and GDC had slight to negligible effects; only PP242 could significantly inhibit cellular growth in some of these cell lines. 

In the case of T47D, ZR75.1 and BT474, all the C2 dominance criteria were confirmed. In addition to p-(Ser473)-Akt, relatively high Rictor expression was also detected in these cells. SKBR3, MDA-MB453 and HS578T cells showed mainly C1 complex activity. Four cell lines were classified into the no complex dominance/balanced mTORC1/C2 category: MCF7 and three TNBC cell lines—MDA-MB231, MDA-MB468 and BT549. MDA-MB468 and BT549 can be considered resistant to mTOR inhibition.

### 2.2. Glycolysis/Warburg Phenotype, Glutaminolysis and Lipid Metabolism in Human Breast Cancer Cell Lines

**Figure 3:**
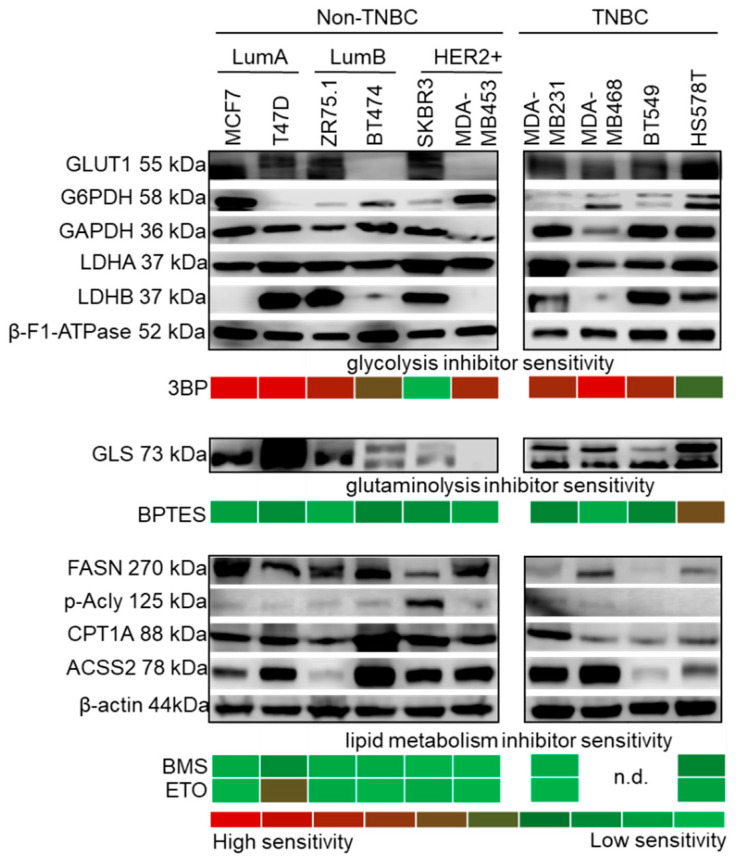
Metabolic characteristics of breast cancer cell lines. Metabolic enzyme expression and inhibitor sensitivity shows individual characteristics in a subtype-independent manner. The upper panel shows representative Western blots of metabolic enzymes indicative of glycolysis and the Warburg effect (GLUT1, G6PDH, GAPDH, LDHA and LDHB), in parallel with 3BP (glycolysis inhibitor, 100 µM) sensitivity. β-F1-ATPase expression was confirmed in all cells. Glutaminase expression (GLS) and the effect of BPTES (GLS1 inhibitor, 10 µM) are shown in the middle panel. The expression of lipid metabolic enzymes (FASN, p-Acly, CPT1A and ACSS2) are shown in the lower panel. The protein profiles of balanced lipid metabolism (with both catabolic and anabolic mechanisms) may account for the resistance to inhibitors of lipid synthesis (BMS, 10 µM) and lipid oxidation (ETO, 50 µM) in the majority of the cell lines. In vitro inhibitor sensitivities are presented with a colour scheme showing inhibitory effects, based on the averages of Alamar Blue and SRB test results: green (0%, resistant cells) to red (~90–100%, highly sensitive cells) (loading control: β-actin.; n.d.: not determined; triple negative breast cancer—TNBC; luminal A—LumA; luminal B—LumB; HER2+ subtypes; inhibitors were tested after 72 h treatment).

The metabolic properties of human breast cancer cell lines were investigated with an approach similar to that for the above experiments: the expression of selected metabolic enzymes as well as sensitivity to specific metabolic inhibitors were determined (Figure 3, Table S1). Interestingly, enzyme expression and metabolic inhibitor sensitivity showed a more complex pattern compared to mTOR pathway data.

3BP (an inhibitor of glycolysis through GAPDH and hexokinase 2—HK2—inhibition) was the most effective treatment in almost all cell lines (Figure 3, Table S1), which highlights the role of in vitro glucose utilisation. Cells expressed considerable amounts of LDHA, whereas LDHB expression was highly variable. Moreover, at least two other enzymes related to glucose utilisation (GLUT1, G6PDH or GAPDH) were also present. However, BT474 and MDA-MB453 cells—which did not express detectable levels of the main glucose transporter GLUT1—had higher sensitivity to 3BP. SKBR3 was the least sensitive to 3BP among the studied cell lines, with an extremely balanced metabolic enzyme profile, including lipolytic, synthetic and glutaminolytic enzymes, without high in vitro glucose addiction (Figure 3). All the TNBC cell lines showed higher LDHA levels compared to LDHB (except for BT549) and were 3BP sensitive, which underlines the importance of the Warburg effect in TNBCs. These cells were more resistant to other treatments, especially MDA-MB468 and BT549 (Figure 3). Considerable β-F1-ATPase expression was detected in each cell line with slight differences, which suggests the presence of functional ATP synthase and OXPHOS.

The GLS1 inhibitor BPTES had a weaker effect. It slightly inhibited the growth of five cell lines (T47D, BT474, SKBR3, MDA-MB231 and BT549), but its inhibitory effect was only significant in HS578T cells (Figure 3). This observation could correlate to GLS expression: the cytotoxicity induced by BPTES was at least 15% in cell lines with detectable amounts of both GLS isoforms (KGA/kidney type GLS and GAC/glutaminase C), except for the highly resistant TNBC cell line—MDA-MB468 (Figure 3).

Lipid synthetic capacity was highly variable in vitro. Increased levels of FASN and/or activated Acly were observed in non-TNBC cells in a cell type-dependent manner. Surprisingly, lipid synthetic proteins were lower in TNBC cell lines; p-Acly expression was undetectable in three TNBC cell lines (Figure 3). Of note, ACSS2—related to acetate consumption—and CPT1A—which provides long-chain FAs for the mitochondria—were present in the majority of the cell lines. This indicates a potentially higher lipid oxidation rate, along with acetate consumption capacity. Since both lipolytic and lipogenic adaptation mechanisms are present in breast cancer cells, the applied lipid metabolism inhibitors had significant antiproliferative effects only in some cell lines: ETO (a CPT1A/lipid oxidation inhibitor) significantly decreased the cellular growth (by 50%) of T47D cells, whereas BMS (an Acly/lipid synthesis inhibitor) slightly inhibited (~20%) the growth of T47D and HS578T cells (Figure 3). 

### 2.3. Metabolic Adaptation in MDA-MB231 Triple-Negative Human Breast Cancer Model

**Figure 4:**
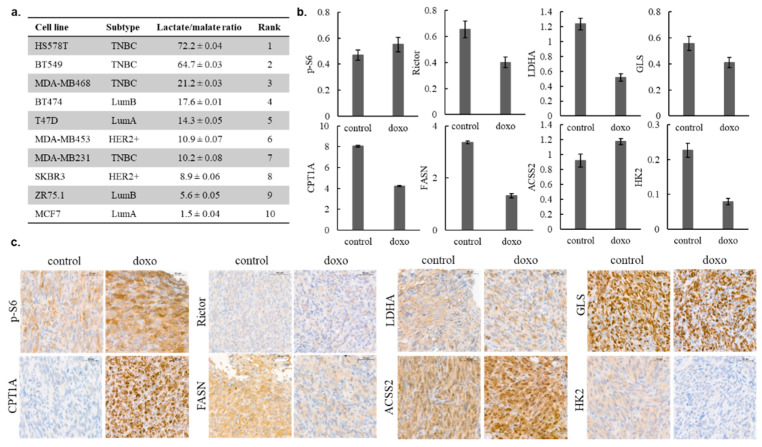
Intracellular lactate–malate ratio as a characteristic of human breast cancer cells; in vitro and in vivo metabolic adaptation of MD-MB231 cells after doxorubicin treatment. Intracellular metabolite concentrations were measured by LC-MS, the lactate–malate ratio was calculated from the ng/10^6^ concentrations, and the cell lines were ranked in accordance with their glycolytic versus oxidative phosphorylation (OXPHOS) shift (**a**); mTOR and metabolic activity-related enzyme levels were assessed by WES Simple, and the protein levels normalised to β-actin were measured in control and in in vitro 72 h doxorubicin-treated (50 ng/mL, doxo) cell lysates (**b**); the same protein-level alterations were studied by immunohistochemistry (IHC) analysis in in vivo doxorubicin (21 days, 2 mg/kg/week)-treated MD-MB231 tumour xenografts (**c**). Scale bars are given in the figures.

To further determine the metabolic characteristics of the studied human breast cancer cell lines, intracellular metabolite concentrations were assessed using liquid chromatography–mass spectrometry (LC-MS). Based on our previous studies, the lactate–malate ratio was calculated to assign differences in glycolytic and OXPHOS capacities. Using this, the cell lines were ranked in accordance with their glycolytic versus OXPHOS shift. The most glycolytic phenotype was characteristic for HS578T, while MCF7 was highly OXPHOS-dependent in our cell culture models (Figure 4a). The MDA-MB231 human TNBC cell line was selected for further investigations based on its overall resistance to the previously presented mTOR and metabolic inhibitors and moderate doxorubicin sensitivity, respectively (doxorubicin induced growth arrest in the studied cell lines, which is shown in Table S1). In relation with the expression profile of the mTOR pathway and other metabolic enzymes, doxorubicin-induced alterations were analysed by the WES Simple technique. In general, the metabolic activity of MDA-MB231 was decreased after 72 h of in vitro treatment, except for the expression of the p-S6 and ACSS2 proteins (Figure 4b). In in vivo xenograft models, 24 days of doxorubicin (50 ng/mL) treatment lead to growth inhibition (tumour sizes were reduced by about 50%; the average tumour weight of the controls was 0.638 ± 0.075 g; the average tumour weight of the doxorubicin-treated group was 0.375 ± 0.107 g; the increases in tumour volumes are given in Figure S2) and almost similar metabolic changes; slightly increased mTORC1, ACSS2 protein levels and, in parallel, decreased levels of other metabolic enzymes were observed using IHC analyses. Only one difference was detected between the in vitro and in vivo data—CPT1A expression was increased in the case of doxorubicin-treated MDA-MB231 tumours in vivo (Figure 4c). These results suggest that metabolic adaptation mechanisms can support the survival of tumour cells during therapeutic treatments.

### 2.4. Clinicopathological Correlation between Selected mTOR Activity and Metabolic Markers in Human Breast Cancer Specimens

According to the above-presented observations, the in situ metabolic features of human breast cancers are in accordance with the obtained in vitro data, which underlines the relevance of the selected markers for performing clinicopathological evaluations.

The expression of p-S6 correlates to mTORC1 activity; in addition, p-(Ser473)-Akt and Rictor expression could be reliable markers for mTORC2 activity in cell lines. However, p-(Ser473)-Akt antibody is not appropriate in biopsy materials (its epitopes can hardly be detected by immunostaining because phosphorylated-Akt proteins rapidly degrade in the tissue microenvironment). Based on our previous and current results, p-S6 and Rictor were selected for the in situ evaluation of human breast cancer samples as priority markers for IHC. Considering the expression patterns of LDHA and GLS, the balance of expression of the proteins characteristic for lipid anabolism and catabolism, and their association with metabolic inhibitor sensitivity, the following markers were selected for IHC: LDHA (to determine Warburg phenotype distribution), GLS (a marker of glutaminolytic capacity) and, in addition, FASN and CPT1A (markers of lipid metabolism).

The applied IHC markers showed characteristic cytoplasmic staining patterns (Figure S3). In line with our previous data, p-S6 and Rictor were detected in the cytoplasm of tumour cells, and no nuclear immunoreactivity was observed. The individual inter-tumoral Rictor staining was variable; however, intra-tumoral heterogeneity (Figure S3d) was detected in about 10% of cases. Similarly to in normal breast ductal epithelial cells (Figure S3f), Rictor was present at detectable levels in tumour tissues (median H-score = 180). Only about 10% of the studied breast cancer cases lacked significant Rictor expression. This suggests that the mTORC2 complex is generally available in both breast ductal epithelium and breast cancer cells.

Compared to normal breast tissues, the majority of the breast carcinomas were characterised by increased p-S6 H-scores (Figure S3b). Additionally, intra-tumoral p-S6 heterogeneity was much higher in breast cancer tissues (Figure S3a). p-S6 staining was also the most heterogeneous of all the examined markers. Among metabolic markers, the expressions of FASN and CPT1A were characterised by higher H-scores in tumour cells (median H-score = 210 and 240, respectively), in contrast to those in normal breast epithelium (Figure S3n,q,o,r, respectively). Based on the functional differences between FASN and CPT1A (FA synthesis vs. oxidation), we expected that high amounts of FASN—and consequently, active lipid synthesis—would exclude high-rate lipid transport to mitochondria. Therefore, FASN and CPT1A stainings would yield an inverse pattern in a given cell or tissue. Surprisingly, our assumption was only partially confirmed (Figure S3m,p). Furthermore, parallel overexpression of FASN and CPT1A was found in more than 60% of cases (Figure S3n,q).

Homogeneous cytoplasmic LDHA and granular/mitochondrial GLS stainings were observed in normal and tumour cells, and the levels of these metabolic enzymes were slightly elevated in tumour tissues. Moderate intra-tumoral and characteristic inter-tumoral heterogeneity for LDHA and GLS were observed in the studied breast cancers (Figure S3g,h,j,k).

To compensate the differences among the incidence and decrease histological variance, the same number of cases was analysed in each breast carcinoma subtype, and only invasive breast carcinoma of no special type (formerly, invasive ductal carcinoma) cases were included in our study. Our results for survival rates corresponded to the expected outcomes using standard treatments and care. 

**Figure 5:**
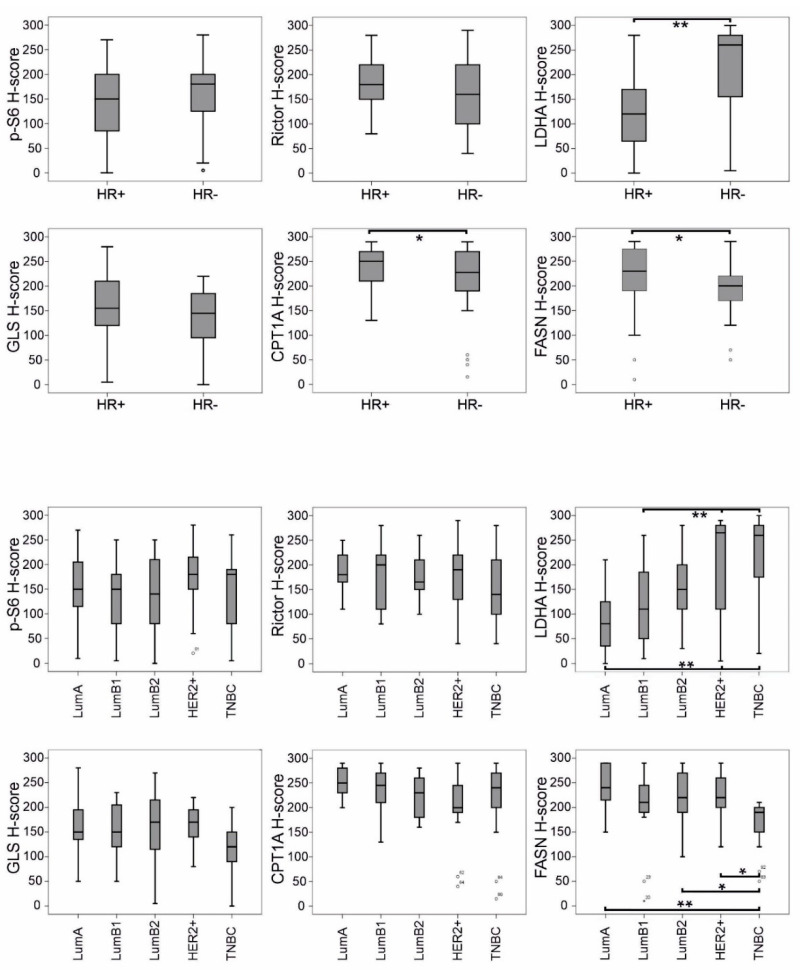
Correlation of mTOR pathway markers and metabolic enzymes (determined by IHC) with subtype distribution in breast cancer. H-scores for LDHA, CPT1A and FASN showed significant correlation with hormone receptor status (HR+ or HR-). LDHA H-scores increased from luminal A (LumA) to triple-negative breast cancer (TNBC) and, conversely, FASN H-scores decreased from LumA to TNBC (** *p* < 0.01, * *p* < 0.05, statistically significant). Ranges with median values are shown; bare circles represent outlier breast cancer cases.

Based on the evaluation of mean H-scores, breast carcinomas expressed p-S6, Rictor and GLS—with the staining patterns described above—independently of HR status (Figure 5). Interestingly, the expression of LDHA and lipid metabolism-related enzymes (CPT1A and FASN) showed an association with HR status: LDHA expression was significantly enhanced in HR- cases, compared with in HR+ ones. As for CPT1A, all breast cancer tissues were characterised by extremely high enzyme expression; however, a slight but significant decrease in H-score was observed in the HR- subtypes—similarly to FASN (Figure 5). According to further refined distinct subtypes, the distribution of LDHA and FASN expression showed an inverse correlation, with LDHA H-scores increasing from LumA to triple-negative breast cancer (TNBC) and, conversely, with FASN H-scores decreasing from LumA to TNBC.

### 2.5. Analysis of In Situ mTOR and Metabolic Protein Expression: Correlation with Breast Cancer Prognosis

Breast cancer surrogate subtype, individual clinical data (including distant metastasis-free survival—DMFS—and overall survival—OS) and the heterogeneity of IHC staining patterns were evaluated to detect further prognostic correlations. Patients were classified into low and high expression categories according to the H-scores of different stainings. Low and high groups were defined using rounded cut-off values based on ROC curve analysis. An H-score of 200 was used as a cut-off value for CPT1A and FASN, while an H-score of 150 was used for the other stainings. 

High p-S6 H-scores positively correlated with distant metastasis and older age in our patient cohort. Furthermore, LDHA expression significantly correlated with subtype distribution, grade and stage (Table 1, Table 2 and Table 3). Some tendencies were observed between clinical data and GLS or FASN expression, but these did not reach statistical significance. 

**Figure 6:**
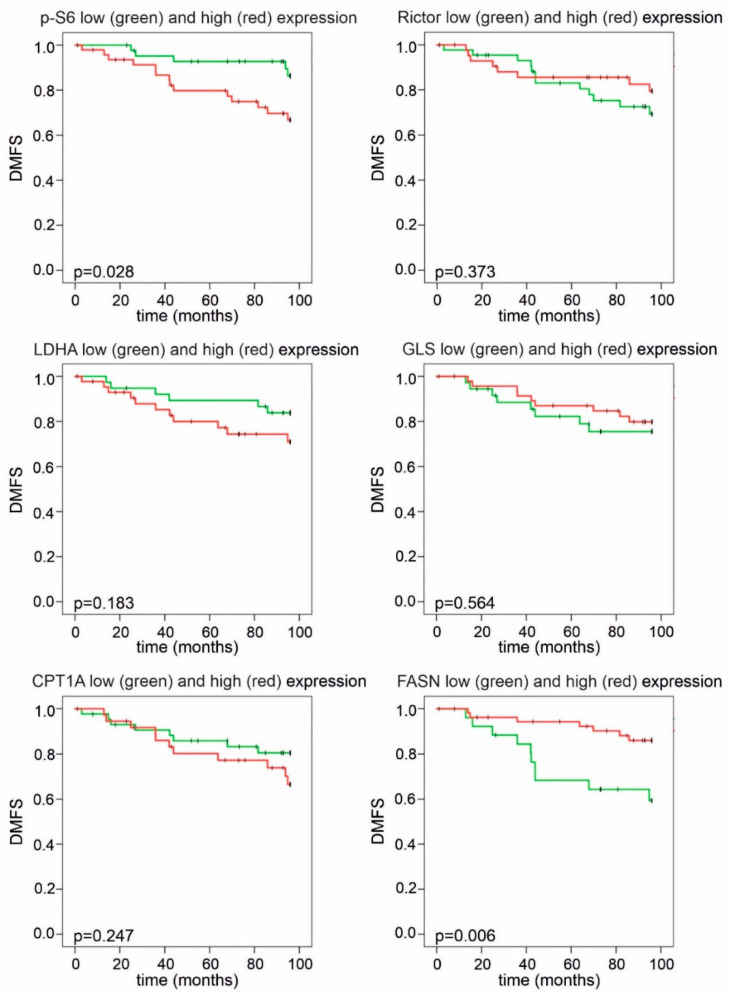
Kaplan–Meier analysis of distant metastasis-free survival in patient groups with high and low IHC scores. Patients with high *p*-S6 or low FASN H-scores had significantly shorter distant metastasis-free survival (DMFS) during a 96-month follow-up. High LDHA H-scores showed a tendency for worse survival. Low and high groups were determined based on median H-scores. The cut-off value was 150 for *p*-S6, LDHA, GLS and Rictor and 200 for FASN and CPT1A expression (*p* < 0.05 was considered statistically significant).

**Figure 7:**
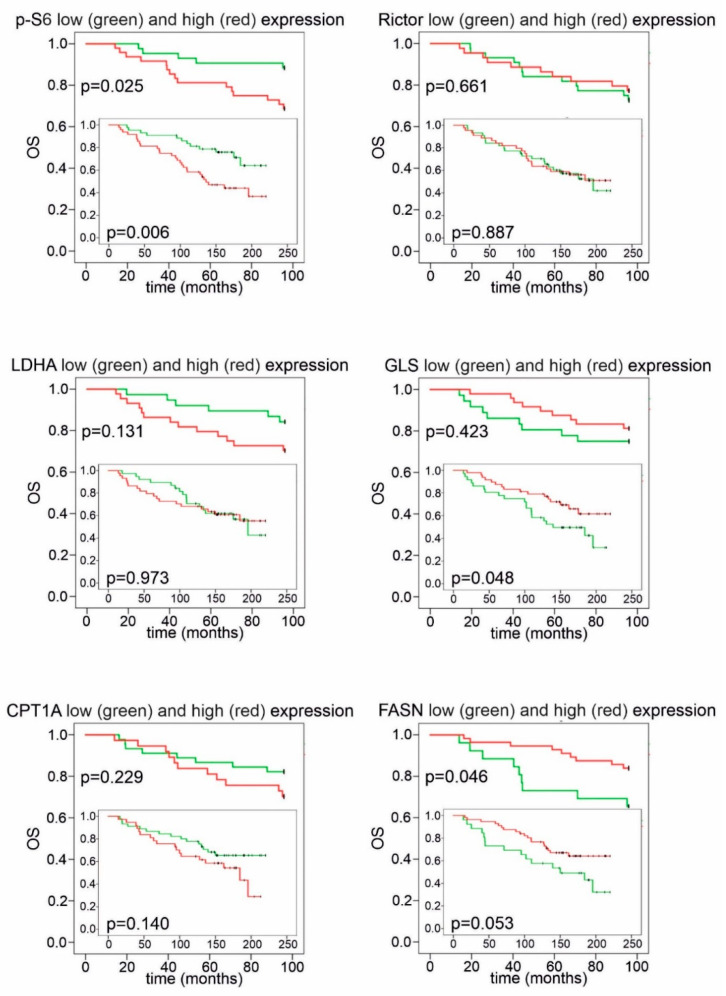
Kaplan–Meier analysis of overall survival in patient groups with high and low IHC scores. High *p*-S6 scores showed a correlation with worse overall survival (OS) during the whole follow-up period. Patients with high GLS scores had better OS, which reached statistical significance during long-term follow-up. High FASN scores indicated significantly better OS in the first 96 months; as for CPT1A, a tendency for worse survival was observed in the group with high H-scores. Low and high groups were determined based on median H-scores. The cut-off value was 150 for *p*-S6, LDHA, GLS and Rictor and 200 for FASN and CPT1A expression. Long-term (>200 months) follow-up data are displayed on insets.

The statistical analysis of DMFS and OS revealed that elevated p-S6 expression had a negative impact on prognosis (Figure 6 and Figure 7). Remarkably, CPT1A and FASN expression divided our patient cohort into two populations, both with distinct survival characteristics and a related inverse pattern of these proteins: high FASN scores significantly correlated with longer DMFS and OS during 96 months of follow-up; conversely, high expression of CPT1A was associated with a tendency for worse prognosis (Figure 6 and Figure 7). Low or high Rictor scores were not associated with survival advantage; however, high GLS expression in breast cancer cells (suggesting potential glutamine consumption) showed a long-term overall survival benefit (Figure 6 and Figure 7).

Meta-analysis of mRNA expression datasets—in silico gene expression analyses—derived from the KM-plotter database supports some of our results for protein expression and survival. High S6, LDHA and CPT1A and, additionally, lower FASN and GLS mRNA expression were associated with worse prognosis in luminal breast cancer types (KM-plotter—Table S2), similarly to our results for survival and the protein-level expression of *p*-S6, LDHA, CPT1A, FASN and GLS in tumour tissues. Survival data related to Rictor and mTOR mRNA differed from our findings. However, protein expression—especially in case of *p*-mTOR—may be more important. Published protein expression data suggest that high mTOR activity (indicated by *p*-mTOR and/or *p*-S6), especially mTORC2 activity (e.g., high levels of Rictor, *p*-(Ser473)-Akt), is an unfavourable prognostic indicator, which is in accordance with our results.

**Figure 8:**
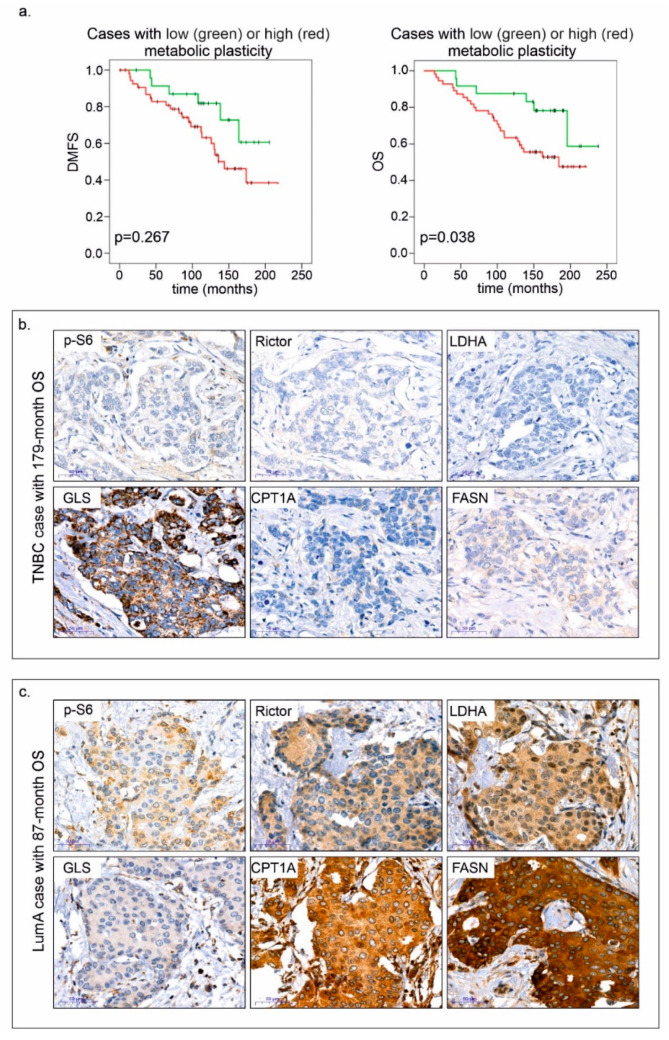
Positive metabolic plasticity correlates with worse prognosis in breast cancer. Kaplan–Meier analysis in patient cohorts associated with negative and positive metabolic plasticity (**a**), and immunostainings—*p*-S6, Rictor, LDHA, GLS, CPT1A and FASN; two representative cases are shown (**b**,**c**). Negative metabolic plasticity and long overall survival (OS—179 months) were observed in a triple-negative breast cancer (TNBC) case (**b**). Conversely, positive metabolic plasticity is exemplified by high H-scores in the majority of proteins including *p*-S6 and Rictor, and shorter overall survival (87 months) in a luminal A (LumA) breast cancer case (**c**). 3:3′-diaminobenzidine (DAB) (brown) was used as a chromogen. Scale bars are given in the figures.

The most interesting observations were derived from multiple profile analyses. By combining mTOR activity scores with the results of other stainings, we could establish two categories: cases associated with negative or positive metabolic plasticity. A high mTOR activity score (high *p*-S6 and/or Rictor H-score) along with high expression of at least two of four metabolic pathway elements (GLS, FASN, CPT1A and LDHA) were required as minimum criteria for positive metabolic plasticity. Patients with tumours showing positive metabolic plasticity had significantly worse prognosis (Figure 8). 

Based on protein expression profiles and clinical data, we aimed to identify factors (specific protein expression or metabolic plasticity scores) that could be beneficial for risk assessment (relative risk—RR) using Cox regression and multivariate analysis. Cox regression analysis (including prognostic variables such as age, subtype, HR status, grade and stage) indicated that high *p*-S6 scores and positive metabolic plasticity could predict a worse outcome (*p*-S6—RR = 2.528; 95% CI, 1.278 to 5.00; *p* = 0.008; plasticity score—RR = 2.48; 95% CI, 1.021 to 6.021; *p* = 0.045). The expression pattern of the other proteins did not have any independent prognostic value in these smaller cohorts, as they were influenced by other factors such as age and additional clinical parameters. Our results highlight the negative impact of high *p*-S6 expression and metabolic plasticity on survival.

## 3. Discussion

Today, both tumour biology and clinical studies show a growing interest in the metabolic properties of tumour cells—including in vivo tissue heterogeneity and metabolic plasticity [25]. Recent transcriptomic and metabolomic analyses suggest that additional complex metabolic phenotypes can be distinguished: beside the well-known glycolytic and OXPHOS phenotypes, novel, hybrid (Warburg + OXPHOS; W/O) and metabolically inactive states should be considered for further studies. These data were obtained primarily from tissue homogenates (from transcriptomic databases) and/or cell culture experiments; however, pathomorphological studies using IHC have rarely been performed [26,27].

The importance of metabolic characterisation was also emphasised by our analysis of tumour tissues and corresponding cell lines—which were used as model systems to both investigate the expression of enzymes involved in metabolic adaptation and the effect of inhibitors targeting the mTOR pathway, glycolysis, glutaminolysis and lipid oxidation/synthesis. As expected, most of the mono-treatments had moderate antiproliferative effects in a subtype-independent manner, which suggests that breast cancer cells have a high level of adaptive capacity and survival/growth potential. Two of the studied cell lines did not express GLUT1; however, these were glycolysis-dependent cells. This underlines the potential importance of other glucose transporters and the glycolysis inhibitor sensitivity of breast cancer cells in vitro [28]. The intense effect of the glycolytic inhibitor (3BP) can be attributed to the in vitro glucose dependence of cell cultures, which is not a universal in vivo feature of breast cancer cells (e.g., PET-CT-negative cases exist [29]). LDHA and LDHB expression (representing Warburg- and reverse-Warburg-effects, respectively) showed individual alterations, displaying the most remarkable differences among glycolytic markers. In parallel with proliferation, we detected increased extracellular acidification in normoxic cell culture conditions. However, both lactate consumption and production were available to serve cellular demand. It is a general observation that pH changes can be indicated (by phenol red) in the medium based on, for example, the increasing amount of lactate in most cell cultures including those of the studied breast cancer cell lines. These observations and the detected high lactate–malate ratio in the majority of the studied in vitro breast cancer cell lines as metabolic characteristics confirm the bioenergetic contribution of the Warburg effect to tumour growth potential, which has been described in several publications on breast cancer, including subtypes with worse prognosis [13,30]. Our results highlight the prognostic importance of the Warburg effect, as LDHA tissue protein expression significantly correlated with subtype distribution, and high H-scores were associated with worse prognosis in the whole studied population after a 96-month follow-up. The inhibition of the Warburg effect and/or glycolysis—including MCTs—has been the focus of numerous cancer-related studies for the last decade. However, except for in some trials, the success lagged behind expectations due to the toxic side-effects or inefficacy (attributed to metabolic adaptation mechanisms) of the tested agents [31].

Targeted mTOR inhibitory therapies are more complex and tumour specific. Rapalogs are introduced in combination with exemestane in advanced oestrogen-receptor-positive breast cancer; moreover, clinical trials suggest the feasibility of mTOR inhibitors in other subtypes such as HER2+ or TNBC [32,33]. The prognostic value of mTOR, *p*-mTOR and other markers of mTOR activity are rather contradictory in different reports [34,35]. Our in situ findings support the observation that high mTORC1 activity (i.e., *p*-S6) correlates with worse prognosis/shorter survival. High *p*-S6 expression may be an independent risk factor in breast cancer, which highlights the importance of mTORC1 hyperactivity in all subtypes. In addition, our data from in vitro treatments and Rictor protein expression studies suggest that high levels of mTORC2 complex proteins is common in cell lines and breast tissues (normal and cancerous). Therefore, the introduction of mTORC1/C2 or dual inhibitors (acting on different levels of the mTOR pathway) should be considered in breast cancer therapy. Addressing this issue, clinical trials have been started and are ongoing. Although side-effects are relatively frequent, they seem to be manageable, and the results are promising [36,37,38]. All these studies emphasise the importance of finding appropriate biomarkers and developing up-to-date combination therapies [36,37,38]. According to the diverse metabolic effects of rapalogs and the individual variability of adaptation/resistance properties, we suggest that other metabolic inhibitors may be able to enhance the effects of mTOR inhibitors and reduce tumour growth. Furthermore, selecting breast cancer cases via mTORC1/C2 complex and metabolic target validation can also be proposed. Experimental studies of highly malignant tumours (e.g., gliomas and hepatocellular carcinomas) indicate that the antitumour effect of rapalogs is significantly enhanced by inhibitors of glycolysis or OXPHOS/mitochondrial biogenesis [14,39]. Additional approaches such as autophagy, lipid oxidation/synthesis or other OXPHOS inhibitors, combined with current targeted therapies, have been successfully tested in malignancies other than breast cancer [40,41,42]. The results of these studies coincide with the observations that metformin treatment lowers, whereas obesity and insulin resistance increase, the incidence of breast cancer [43,44]. 

Among targeting strategies, glutaminolysis and lipid metabolism cannot be neglected, as they serve as potential metabolic switches. The diverse bioenergetic consumption processes for glutamine (oxidation or reductive carboxylation) contribute to high adaptive capacity, which has been observed in a number of malignancies [21,24,45]. The glutamine-converting enzymes of these mechanisms are the glutaminases (including two isotypes—GLS1 and GLS2—and their isoforms), which play an important regulatory role in metabolic adaptation [45]. The regulatory roles of GLS isoforms can be different, and their expression patterns can also influence these processes. The lower-molecular-weighted GAC isoform (also present in our cell lines) was reported to have substantial tumorigenic potential [46]. In almost all the studied cases, the tissue expression of GLS was high; however, high GLS H-scores were associated with better survival in our patient cohort. This contradiction may be explained by the fact that the isoforms cannot be differentiated in situ due to the lack of appropriate antibodies, and the low effectivity of GLS1 inhibitor mono-therapy might be attributed to low amounts of GAC. These assumptions need to be further elucidated. Fuelling the TCA (tricarboxylic acid) cycle from glutaminolysis or replenishing acetyl coenzyme A (ac-CoA) can be supported by several rapidly reactivated pathways, depending on cell type and tissue environment, especially in tumour tissues [19]. The alteration of lipid metabolism is characteristic for many different cancers. The overexpression of FASN can predict a poor prognosis in gastric and prostate cancers [47]. Both lipolytic and liposynthetic enzymes are present in the studied breast cancer cells in vitro. Based on our findings, this feature can also be present in breast cancer cell populations in situ in a subtype-independent manner. CPT1A and FASN expression was considerable in the majority of tissue samples. In fact, CPT1A and FASN were simultaneously co-expressed in two thirds of our cases, which indicates the potential of enhancing adaptive mechanisms in lipid metabolism. Surprisingly, FASN overexpression has some association with better prognosis in the investigated cases. We could underline the importance of this finding with some new published data from Kim S et al. [48]. They described that FASN overexpression correlated with better outcome in progesterone-negative cases of breast cancer patients. In our progesterone-negative patient cohort, FASN overexpression slightly correlated with better DMFS, but this was not statistically significant, unfortunately. Furthermore, high CPT1A H-scores and low H-scores of lipid-synthetic FASN correlated with worse prognosis according to survival analysis. Our results suggest that the potentially increased mitochondrial transfer of long-chain FAs may support malignant clone selection and predict worse prognosis in primary breast cancer. In line with our findings, lapatinib-resistant cells were reported to upregulate CD36 (FA transporter) expression to enhance exogenous FA uptake [49], which underlines the role of in situ alterations in lipid metabolism in breast cancer progression. 

The inhibition of the above-discussed processes is not effective enough; however, combinations with other metabolic inhibitors or targeted therapies may improve treatment outcomes. For instance, RAPA may synergise with FASN inhibitors, which induces cytotoxicity in ER/HER2+ breast cancer cells [50]. It is well-known that tumour stemness and dormancy support tumour cell survival and reactivation in breast cancer. The switch between lipid oxidation and synthesis may have special roles in such adaptation. These provide new options [23,51,52] to target specific metabolic rearrangements of potentially surviving chemoresistant cells. These metabolically rewired stem cells—such as cells that undergo epithelial–mesenchymal transformation or “energetic” cancer stem cells—possess several adaptation mechanisms, which might be the “Achilles heel” of cancer [53,54]. Our in vitro and in vivo results about metabolic and mTOR activity alterations in MDA-MB231 model systems include the following: (a) the expression of mTORC1 and ACSS2 is increased and other metabolic enzymes’ expression is mainly decreased in doxorubicin-treated MDA-MB231 cells in vitro, and (b) mTORC1 activity and ACSS2 protein expression are similarly increased; moreover, CPT1A is elevated in vivo in MDA-MB231 xenografts as a sign of metabolic shift during doxorubicin chemotherapy, highlighting the importance of metabolic alterations at the tissue level. In addition, our findings that mTOR signalling-activity-related markers (both *p*-S6 and Rictor expression), as part of metabolic plasticity evaluation, help to predict prognosis subtype independently underline the importance of metabolic rewiring mechanisms in tumour cells and tissues, as well. 

**Figure 9:**
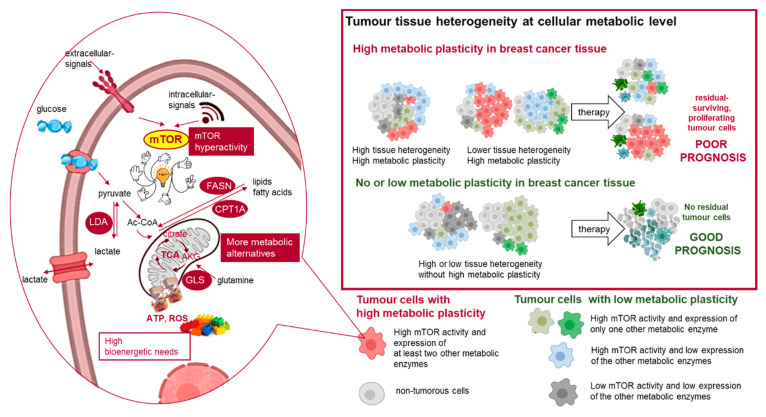
Metabolic adaptation in breast cancer tissues and their impact on therapy response. The individual differences in cellular metabolic phenotype contribute to tissue heterogeneity in the tissue microenvironment. The schematic figure summarises the examined elements of metabolic adaption and the detected different metabolic subtypes in the studied breast cancer cells (cells with high metabolic plasticity: high mTOR activity and high expression of at least two other studied metabolic enzymes; cells with no or low metabolic plasticity: low mTOR activity or high mTOR activity and high expression of only one studied metabolic enzyme). LDHA (lactate dehydrogenase A) is involved in glycolysis, GLS (glutaminase) is involved in glutaminolysis, and FASN (fatty acid synthase) and CPT1A (carnitine palmitoyltransferase 1A) are involved in lipid metabolism.

The results of our group and other groups emphasise that metabolic plasticity and the presence of hybrid metabolic phenotypes are key players in developing therapy resistance [55,56]. The novelty of our work is that we investigated several metabolic pathways and tissue profiles simultaneously. We showed that multiple metabolic rewiring with concomitant mTOR hyperactivity is an unfavourable prognostic sign in breast cancer independent of subtype. The evolution of tissue heterogeneity, metabolic diversity and plasticity needs to be further elucidated in treatment-naive and treated patients for the development of novel, cutting-edge therapies (Figure 9). However, the increased severity of the side-effects of metabolic inhibitors also have to be considered, and these necessitate special patient follow-up and care during clinical assistance.

## 4. Methods

All materials were purchased from Sigma-Aldrich, except where otherwise indicated in the text.

### 4.1. Cell Cultures and In Vitro Treatments 

Ten human breast cancer cell lines were purchased from ATCC (Table S3) and used for in vitro experiments (LumA: MCF7 and T47D; LumB: ZR75.1 and BT474; HER2+: SKBR3 and MDA-MB453; TNBC: MDA-MB231, MDA-MB468, BT549 and HS578T). Based on literature data and the COSMIC and Sanger databases, the most frequent oncogenic mutations were collected, and these are shown in Table S3 (see also [57]). Cells were cultured in RPMI 1640 (2 g/L glucose; Biosera, Nuaille, France), DMEM high glucose (4.5 g/L glucose; Biosera; for HS578T) or DMEM low glucose (1.5 g/L glucose; Biosera; for SKBR3) media supplemented with 10% foetal bovine serum (FBS; Biosera) and gentamycin (Sandoz, Basel, Switzerland; 80 mg/2 mL) or 100 UI/mL penicillin–streptomycin (Biosera; for SKBR3) and 2 mM L-glutamine (Biosera; 4 mM for HS578T). The cells were seeded into T25 flasks (for Western blots/WES) or plated onto 96-well plates (for Alamar Blue and sulforhodamine B (SRB) tests) at a cell density of 3–8 × 10^5^ cells/flask or 2–5 × 10^3^ cells/well, depending on the type of experiment and cell line. Cells were treated with RAPA (50 ng/mL), PP242 (1 μM; Tocris Bioscience, Bristol, UK), BMS-303141 (BMS; 10 μM; fatty acid synthesis inhibitor), etomoxir (ETO; 50 µM), 3-bromopyruvate (3BP; 100 µM), bis-2-(5-phenylacetoamido-1,3,4-thiadiazol-2-yl)-ethyl sulphide (BPTES; 10 μM), GDC0068 (GDC; 1 μM; Cayman Chemical, Ann Arbor, MI, USA) and doxorubicin (doxo; 50 ng/mL) for 72 h (for additional information, see Table S4). DMSO (1%) was used as a vehicle and was added to control cells as required. Concentrations were determined and used based on previous experiments and publications [40].

### 4.2. In Vitro Toxicity Assays

The metabolic activity and protein contents of the cell cultures were analysed by Alamar Blue (Thermo Fisher Scientific, Waltham, MA, USA) and SRB assays after treatments, respectively. The cells were incubated with Alamar Blue for 4 h, and the fluorescence was measured with a Fluoroskan Ascent FL fluorimeter (Labsystems International; Ascent Software, Vantaa, Finland) at 570–590 nm. For the SRB assay, the cells were fixed with 10% trichloroacetic acid for 1 h and incubated with SRB (0.4 *m*/*v*%) for 15 min. Tris base (10 mM) was used to re-solubilise the protein-bound dye. The absorbance was measured at 570 nm using a Multiskan MS microplate reader (Labsystems International; Transmit Software, Vantaa, Finland). At least three independent experiments with a minimum of six parallels were performed for each treatment. The percentage of cytotoxicity caused by the used inhibitors/therapeutic agents is given relative to control samples.

### 4.3. Expression Analysis of Proteins by Western Blot Analyses or WES Simple on Samples of Human Breast Cancer Cell Lines

The protein concentrations of cell extracts (~10^6^ cells) were quantitated with the Bradford assay (Bio-Rad, Hercules, CA, USA). Proteins were separated by SDS-PAGE and transferred to PVDF membranes using a wet blotting system (Bio-Rad). The membranes were incubated with the primary antibodies and anti-β-actin as a loading control. Vectastain Elite ABC HRP Kit (Vector) was used as a secondary antibody. The blots were developed with enhanced chemiluminescence (Thermo Fisher ECL Western Blotting Substrate) and visualised with a C-Digit Blot Scanner (LI-COR Biotechnology, Lincoln, NE, USA). 

WES Simple (ProteinSimple 004-600, Minneapolis, MN, USA) analysis was also performed. A 12–230 kDa Separation Module (ProteinSimple SM-W004) was used for all the proteins, and either the Anti-Rabbit Detection Kit (ProteinSimple DM-001) or Anti-Mouse Detection Kit (ProteinSimple DM-002) were used, depending on the primary antibodies. Briefly, based on the used primary antibodies, 0.2 or 1 μg/μL cell lysates were diluted in 0.1× WES Sample Buffer (ProteinSimple 042-195), and Fluorescent Master Mix (1:4, ProteinSimple PS-FL01-8) was also added. After incubation (95 °C, 5 min), the Antibody Diluent (ProteinSimple 042-203), the primary and secondary antibodies and the chemiluminescent substrate were applied to the WES capillary plate. The WES system settings were (a) stacking and separation (395 V, 30 min), (b) blocking (5 min), (c) incubations with primary and secondary antibodies (30 min) and (d) luminol/peroxide chemiluminescence detection (15 min) (the exposure time was 2 s). The electropherograms were manually corrected if required for the evaluations.

The primary antibodies and their dilution for the Western blot analysis and WES Simple technique are summarised in Table S5. 

### 4.4. Intracellular Metabolite Concentration Measurement Using Liquid Chromatography–Mass Spectrometry

Intracellular lactate and malate were extracted by a modified method based on Szoboszlai et al. [58]. In brief, metabolites were extracted from cells with methanol–chloroform–water (9:1:1). After centrifugation (15,000× *g*, 10 min, 4 °C), the supernatants were stored at −80°C until the measurements. The range of the concentrations of lactate and malate was assessed by using calibration curves. The dilution of the analytical grade standards was 0.5–50 µM, and cell concentrations were calculated as ng/10^6^ cells in ratios. An Agilent 1100 Series high-performance liquid chromatography system coupled with a Sciex QTRAP 6500 mass spectrometer was used for liquid chromatography–mass spectrometry (LC-MS) analyses. A Halo Penta-HILIC column (150 × 2.1 mm, 2.7 µm) (Hichrom Ltd., Berkshire, UK) was applied to perform chromatographic separation. The mobile phase A was 10 mM NH_4_FA at pH = 5, and mobile phase B was acetonitrile containing 0.1% (*v*/*v*) formic acid. The MS was operated in negative electrospray ionization mode. The setup of the measurements was the following: source temperature: 450 °C ionization voltage: −4500 V; entrance potential: −10 V; curtain gas: 45 psi; gas1: 45 psi; gas2: 35 psi; CAD gas: medium. Quantitative analyses were carried out using multiple reaction monitoring (MRM) mode.

### 4.5. Doxorubicin Treatment of MDA-MB231 Xenografts

Xenograft tumours were established in SCID mice by injecting MDA-MB231 cells (2.5 × 10^6^) subcutaneously into the breast region of 8–10-week-old mice. When the tumours were palpable, the animals were divided into control and doxorubicin-mono-treated groups (*n* = 10 each group). The controls were treated with saline. Doxorubicin (TEVA, intravenously, at 2 mg/kg body weight) was administered once per week for 24 days. Body weight and tumour size were registered. Tumour volume was calculated as following: 0.52 × (shorter diameter)^2^  +  longer dimaeter. At the end of the experiments, the tumour weights of the euthanized animals were measured, and the tumour tissues were formalin-fixed and paraffin-embedded. In vivo experiments were approved by the Institutional Animal Care Facility and the Institutional Ethical Review Board (PEI/001/2457-6/2015) with official permissions (PEI/001/1733-2/2015).

### 4.6. Immunohistochemistry on Human Tissues

Formalin-fixed paraffin-embedded (FFPE) breast cancer (LumA, LumB1, LumB2, HER2+ and TNBC, *n* = 15 for each subtype) and normal (non-malignant) breast tissues obtained after breast reduction surgery (*n* = 4) were analysed. Tumour subtypes were defined based on the recommendations of the 13th St Gallen International Breast Cancer Conference [59]. Tumours and normal tissues were obtained by surgical resection at the 1st Department of Surgery, Semmelweis University (Budapest, Hungary) between August 1999 and December 2019. Pathological analysis was performed at the 2nd Department of Pathology, Semmelweis University (Budapest, Hungary). All the tumours were primary lesions, and none of the patients received treatment prior to surgery. Clinicopathological data were obtained from medical records. In our study, the involved cases were diagnosed more than 8 years ago, and the follow-up period was at least 15 years in about 65% of the cases, which enabled us to examine long-term outcomes. Distant metastasis-free survival (DMFS) was assessed and defined as the time elapsed between the first diagnosis of primary breast carcinoma and the date of the diagnosis of a distant metastasis. Archived tissue samples and patient follow-up data were used with the approval of the Hungarian Scientific Council National Ethics Committee for Scientific Research (No. 7/2006). 

For immunohistochemistry (IHC), endogenous peroxidase activity was blocked on deparaffinised tissue sections, and antigen retrieval was performed with citrate buffer (pH = 6) in a pressure cooker. Antibodies against *p*-S6, Rictor, GLS, LDHA, CPT1A, FASN, ACSS2 and HK2 were used for 90 min at room temperature or at 4 °C overnight. Primary antibodies and their sources and dilutions are listed in Table S5. The primary antibodies were detected with Novolink Polymer Detection Systems (Leica) or a Vectastain Elite ABC kit (Vector Laboratories Inc.) using DAB (Dako) as a chromogen. Slides were counterstained with haematoxylin. We aimed to determine the staining characteristics of different mTOR and metabolic markers. The manual assessment of histo-scores (H-scores) was performed by two independent pathologists. In case of disagreement over scoring, a third observer was also involved in the evaluation. An H-score (range: 0–300) was calculated by multiplying the staining intensity (0–3+) by the percentage of positive cells for each intensity [60]. We defined staining patterns as heterogeneous in those cases, where tumour cells were labelled with three different staining intensities simultaneously (+, ++ and +++); additionally, none of the intensity percentages reached 50% in the investigated specimens.

### 4.7. mRNA Expression Data from the KM-Plotter Database

The association between mRNA expression related to mTOR or metabolic pathways and prognosis was estimated using the Kaplan–Meier Plotter database (KM-plotter; http://www.kmplot.com). This online, open-access survival analysis tool was used to query publicly available microarray data from up to 3951 breast cancer patients [61]. Using this, we could determine the prognostic value of previously published mRNA expression data. Hazard ratios were calculated at the best auto-selected cut-off; *p*-values were evaluated using the log-rank test and plotted in R. 

### 4.8. Statistical Analysis

The IBM SPSS Statistics software (version: 22; SPSS Inc., Chicago, IL, USA was used to perform statistical analysis. Mean values and SDs were calculated from three independent experiments with at least six parallels, depending on the used in vitro assays. The Mann–Whitney U-test, Fisher’s test, the log-rank test and Kaplan–Meier analysis were applied to compare the expression of protein IHC markers and survival. Low and high categories (for tissue protein H-scores) were defined using rounded cut-off values based on ROC curve analysis. Hazard ratios were calculated by the Cox regression model for the multivariate analysis of protein expression and other factors. Student’s *t* test was also used for statistical calculations. *p* ≤ 0.05 was considered statistically significant.

## 5. Conclusions

Using markers of the mTOR pathway and cellular metabolism, we identified 1. the distribution of mTOR activity; 2. potential metabolic plasticity (due to inducible metabolic pathways), leading to therapy resistance; 3. patient cohorts with potentially worse prognosis, even in subtypes generally considered low-risk; and 4. potential metabolic targets for future personalised therapy. In our pathomorphological work, breast cancer tissues were investigated in a complex manner to reveal metabolic diversity. We showed the prognostic potential of characterising metabolic pathways (e.g., the glucose-dependent Warburg effect, OXPHOS, glutamine and lipid metabolism) using a set of immunoreactions in breast cancer tissues. Metabolic rewiring is indeed emerging as a promising target, which should be considered in the context of other pathways, such as autophagy. Metabolic heterogeneity can contribute to unfavourable prognosis and could be targeted by combining antimetabolic drugs with mTOR inhibitors or recent therapies. We suggest scoring cellular metabolic variability, which may serve as a baseline for drug development and future treatment design.

## Figures and Tables

**Table 1:** Correlation of clinicopathological parameters of breast cancer patients with selected markers of mTOR complexes (determined by IHC). * *p* < 0.05.

	*p*-S6 Expression				Rictor Expression			
	“Low”	“High”	95% CI	*p* Value	“Low”	“High”	95% CI	*p* Value
Age (years)								
≤55 (*n* = 41)	25 (61)	16 (39)	105.26–150.84	0.021 *	17 (46)	20 (54)	157.00–196.51	0.666
>55 (*n* = 54)	18 (36)	32 (64)	147.23–186.97		27 (53)	24 (47)	153.35–188.61	
Subtype								
	*n* = 43	*n* = 48		0.733	*n* = 44	*n* = 44		0.613
Luminal A (*n* = 16)	7 (47)	8 (53)	103.10–196.90		6 (40)	9 (60)	167.88–213.45	
Luminal B1 (*n* = 19)	10 (56)	8 (44)	103.85–167.81		9 (47)	10 (53)	142.67–205.75	
Luminal B2 (*n* = 22)	12 (55)	10 (45)	106.69–172.40		10 (56)	8 (44)	154.70–204.19	
HER2+ (*n* = 19)	7 (37)	12 (63)	135.85–207.47		7 (41)	10 (59)	135.10–211.96	
TNBC (*n* = 19)	7 (41)	10 (59)	111.64–188.36		12 (63)	7 (37)	121.35–184.97	
Grade				0.256				0.441
1 (*n* = 7)	3 (43)	4 (57)	78.66–218.48		4 (67)	2 (33)	125.89–224.11	
2 (*n* = 38)	13 (37)	22 (63)	140.59–188.55		15 (43)	20 (57)	168.06–205.09	
3 (*n* = 50)	27 (55)	22 (45)	117.37–160.38		25 (53)	22 (47)	143.90–182.91	
pT Stage				0.146				0.636
1 (*n* = 44)	25 (58)	18 (42)	122.80–165.80		17 (43)	23 (57)	163.11–199.89	
2 (*n* = 40)	15 (39)	23 (61)	128.96–180.52		21 (57)	16 (43)	147.20–187.93	
3 (*n* = 5)	2 (50)	2 (50)	13.11–216.89		3 (60)	2 (40)	91.72–220.28	
4 (*n* = 6)	1 (17)	5 (83)	100.80–252.54		3 (60)	2 (40)	71.33–268.67	
pN Stage				0.596				0.829
x (*n* = 1)	0 (0)	1 (100)	not applicable		1 (100)	0 (0)	not applicable	
0 (*n* = 42)	20 (50)	20 (50)	121.27–169.23		17 (45)	21 (55)	158.09–199.28	
1 (*n* = 38)	16 (44)	20 (56)	135.20–182.03		20 (56)	16 (44)	150.11–191.56	
2 (*n* = 12)	7 (58)	5 (42)	70.88–164.95		5 (45)	6 (55)	132.30–231.16	
3 (*n* = 2)	0 (0)	2 (100)	0.00–300.00		1 (50)	1 (50)	0.00–300.00	
Metastasis (*n* = 38)	8 (23)	27 (77)	141.92–189.23	0.0003 *	21 (58)	15 (42)	144.52–186.59	0.278
W/O Metastasis (*n* = 57)	35 (63)	21 (37)	119.47–159.46		23 (44)	29 (56)	162.10–195.59	

pT: pathologic T stage (tumour size); pN: pathologic N stage (lymph node metastases); W/O: without.

**Table 2:** Correlation of clinicopathological parameters of breast cancer patients with GLS and LDHA expression (determined by IHC). * *p* < 0.05.

	GLS Expression				LDHA Expression			
	“Low”	“High”	95% CI	*p* Value	“Low”	“High”	95% CI	*p* Value
Age (years)								
≤55 (*n* = 41)	19 (53)	17 (47)	120.64–157.97	0.125	20 (56)	16 (44)	118.18–181.18	0.182
>55 (*n* = 54)	17 (35)	31 (65)	145.22–180.20		18 (39)	28 (61)	140.57–196.17	
Subtype								
	*n* = 36	*n* = 48		0.296	*n* = 38	*n* = 44		0.001 *
Luminal A (*n* = 16)	4 (29)	10 (71)	145.67–204.33		11 (79)	3 (21)	50.42–126.73	
Luminal B1 (*n* = 19)	8 (42)	11 (58)	122.41–177.59		11 (69)	5 (31)	79.16–163.34	
Luminal B2 (*n* = 22)	8 (42)	11 (58)	123.65–195.83		8 (47)	9 (53)	117.49–190.75	
HER2+ (*n* = 19)	5 (33)	10 (67)	138.86–187.80		5 (31)	11 (69)	149.03–254.09	
TNBC (*n* = 19)	11 (65)	6 (35)	94.42–145.58		3 (16)	16 (84)	174.95–258.74	
Grade				0.118				0.0002 *
1 (*n* = 7)	1 (14)	6 (86)	141.73–229.70		6 (100)	0 (0)	6.26–107.07	
2 (*n* = 38)	12 (36)	21 (64)	141.17–185.19		20 (61)	13 (39)	104.10–155.29	
3 (*n* = 50)	23 (52)	21 (48)	122.66–156.43		12 (28)	31 (72)	169.65–226.86	
pT Stage				0.183				0.024 *
1 (*n* = 44)	12 (32)	25 (68)	157.23–192.50		22 (58)	16 (42)	102.66–164.44	
2 (*n* = 40)	18 (49)	19 (51)	119.88–156.33		13 (37)	22 (63)	154.45–215.84	
3 (*n* = 5)	2 (40)	3 (60)	126.44–197.56		3 (75)	1 (25)	0.00–300.00	
4 (*n* = 6)	4 (80)	1 (20)	0.00–192.14		0 (0)	5 (100)	173.80–246.20	
pN Stage				0.328				0.396
x (*n* = 1)	0 (0)	1 (100)	not applicable		1 (100)	0 (0)	not applicable	
0 (*n* = 42)	13 (36)	23 (64)	128.99–176.29		17 (45)	21 (55)	129.80–186.25	
1 (*n* = 38)	15 (44)	19 (56)	139.45–172.90		17 (53)	15 (47)	116.32–189.93	
2 (*n* = 12)	6 (55)	5 (45)	108.82–178.45		2 (22)	7 (78)	141.79–273.76	
3 (*n* = 2)	2 (100)	0 (0)	0.00–227.06		1 (50)	1 (50)	0.00–300.00	
Metastasis (*n* = 38)	15 (45)	18 (55)	126.25–171.02	0.822	13 (39)	20 (61)	143.85–211.91	0.369
W/O Metastasis (*n* = 57)	21 (41)	30 (59)	139.39–171.20		25 (51)	24 (49)	122.69–174.25	

pT: pathologic T stage (tumour size); pN: pathologic N stage (lymph node metastases); W/O: without.

**Table 3:** Correlation of clinicopathological parameters of breast cancer patients with CPT1A and FASN expression (determined by IHC). *p* < 0.05.

	CPT1A Expression				FASN Expression			
	“Low”	“High”	95% CI	*p* Value	“Low”	“High”	95% CI	*p* Value
Age (years)								
≤55 (*n* = 41)	18 (51)	17 (49)	212.97–249.60	0.657	11 (30)	26 (70)	185.67–224.06	0.814
>55 (*n* = 54)	27 (57)	20 (43)	207.78–239.46		15 (33)	30 (67)	197.95–234.05	
Subtype								
	*n* = 45	*n* = 37		0.454	*n* = 26	*n* = 56		0.208
Luminal A (*n* = 16)	6 (40)	9 (60)	238.20–271.13		3 (20)	12 (80)	216.35–267.65	
Luminal B1 (*n* = 19)	9 (50)	9 (50)	208.35–252.76		5 (31)	11 (69)	162.74–243.51	
Luminal B2 (*n* = 22)	9 (53)	8 (47)	202.80–247.79		6 (30)	14 (70)	195.61–243.39	
HER2+ (*n* = 19)	11 (73)	4 (27)	162.57–240.76		3 (20)	12 (80)	198.33–248.33	
TNBC (*n* = 19)	10 (59)	7 (41)	190.01–254.69		9 (56)	7 (44)	141.00–194.00	
Grade				1.000				0.119
1 (*n* = 7)	3 (50)	3 (50)	193.81–292.85		0 (0)	6 (100)	220.25–263.09	
2 (*n* = 38)	19 (56)	15 (44)	214.55–244.87		14 (41)	20 (59)	179.16–230.25	
3 (*n* = 50)	23 (55)	19 (45)	203.07–241.45		12 (29)	30 (71)	196.68–226.65	
pT Stage				0.625				0.154
1 (*n* = 44)	19 (53)	17 (47)	215.93–249.90		8 (21)	31 (79)	202.23–239.82	
2 (*n* = 40)	20 (56)	16 (44)	210.03–244.41		14 (41)	20 (59)	180.26–222.09	
3 (*n* = 5)	2 (40)	3 (60)	107.99–300.00		2 (40)	3 (60)	150.92–273.08	
4 (*n* = 6)	4 (80)	1 (20)	129.45–238.55		2 (50)	2 (50)	76.99–300.00	
pN Stage				0.145				0.186
x (*n* = 1)	0 (0)	1 (100)	not applicable		0 (0)	1 (100)	not applicable	
0 (*n* = 42)	24 (67)	12 (33)	205.83–238.34		13 (34)	25 (66)	181.63–223.11	
1 (*n* = 38)	16 (48)	17 (52)	210.70–249.30		8 (24)	26 (76)	197.75–238.14	
2 (*n* = 12)	5 (50)	5 (50)	168.28–273.72		3 (43)	4 (57)	179.18–257.96	
3 (*n* = 2)	0 (0)	2 (100)	142.94–300.00		2 (100)	0 (0)	not applicable	
Metastasis (*n* = 38)	16 (50)	16 (50)	198.99–243.51	0.503	12 (38)	20 (62)	172.64–220.49	0.467
W/O Metastasis (*n* = 57)	29 (58)	21 (42)	216.92–244.08		14 (28)	36 (72)	205.38–235.02	

pT: pathologic T stage (tumour size); pN: pathologic N stage (lymph node metastases); W/O: without.

## Data Availability

The authors declare that the data supporting the findings of the presented study are available within the article.

## Supplementary Materials

The following are available online at https://www.mdpi.com/2072-6694/12/9/2492/s1. Figure S1: Additional information on Western blots and WES Simple. (a) The uncropped Western blot bands and densitometric values of the blots related to Figure 2. (b) The uncropped Western blot bands and densitometric value of the blots related to Figure 3. (c) The WES Simple electropherograms of the studied proteins related to Figure 4. Figure S2: The growth curves of the in vivo MDA-MB231 xenografts after doxorubicin treatment. Doxorubicin treatment (2 mg/kg) reduced the growth and tumour volume (calculated by 0.52 × (shorter diameter)2 × longer diameter) of MDA-MB231 in vivo. Figure S3: The expression of selected protein markers in human breast cancer tissues. IHC analysis of p-S6 (a–c), Rictor (d–f), LDHA (g–i), GLS (j–l), FASN and CPT1A in formalin-fixed paraffin-embedded human tissues (breast cancer—a,b,d,e,g,h,j,k,m,n,p,q—and normal tissues—c,f,e,o,r); heterogenous stainings and homogenous stainings were also shown in the first and second columns, respectively. The intra-tumoral heterogeneity of Rictor is labelled (dotted—low staining intensity; full circle—high staining intensity). Representative photos are shown. DAB (brown) was used as a chromogen. H-scores related to specific cases and scale bars are indicated. Table S1: Average results from Alamar Blue and SRB tests in human breast cancer cell lines. Table S2: KM-plotter meta-analysis of mRNA expression and clinicopathological data from breast cancer studies. Table S3: List of breast cancer cell lines, including their IDs, most important mutations and subtypes. Table S4: List of the applied mTOR and metabolic inhibitors. Table S5: List of primary antibodies used for Western blotting and immunohistochemistry.

## Abbreviations

ACSS2	acyl-coenzyme A synthetase short-chain family member 2
Akt	protein kinase B
BPTES	bis-2-(5-phenylacetoamido-1,3,4- thiadiazol-2-yl)-ethyl sulphide
CDK4/6	cyclin-dependent kinase 4 and 6
CPT1A	carnitine palmitoyltransferase 1A
CST	Cell Signaling Technology
DAB	3:3′-diaminobenzidine
DMFS	Distant metastasis-free survival
DMSO	dimethyl sulfoxide
ECL	enhanced chemiluminescence
EGFR	epidermal growth factor receptor
FA	fatty acid
FASN	fatty acid synthase
FBS	foetal bovine serum
FFPE	formalin-fixed paraffin-embedded
G6PDH	glucose-6-phosphate dehydrogenase
GAC	glutaminase C
GAPDH	glyceraldehyde 3-phosphate dehydrogenase
GLUT1	glucose transporter 1
GLS	glutaminase
HER2	human epidermal growth factor receptor 2
HK2	hexokinase 2
HR	hormone receptor
HRP	horseradish peroxidase
IHC	immunohistochemistry
KGA	kidney type glutaminase
LDHA	lactate dehydrogenase A
LDHB	lactate dehydrogenase B
MCT	monocarboxylate transporter
mTOR	mammalian target of rapamycin
OS	overall survival
OXPHOS	oxidative phosphorylation
PARP	poly (ADP-ribose) polymerase
PD-1	programmed cell death protein 1
PD-L1	programmed death-ligand 1
PI3K	phosphatidylinositol 3-kinase
PI3KCA	phosphatidylinositol 3-kinase catalytic subunit A
PKM2	pyruvate kinase 2
PVDF	polyvinylidene difluoride
RR	relative risk
S6K1	ribosomal protein S6 kinase beta-1
SRB	sulforhodamine B
SREBP1	sterol regulatory element-binding protein 1
TCA	tricarboxylic acid
TNBC	triple-negative breast cancer
VEGF	vascular endothelial growth factor
β-F1-ATPase	β-F1-adenosine triphosphate synthase
