# Supplementary material for: In Situ Metabolic Characterisation of Breast Cancer and Its Potential Impact on Therapy
Source: Cancers (Basel). 2020 Sep 3;12(9):2492. doi: 10.3390/cancers12092492 (PMC7563878; doi:10.3390/cancers12092492)
Supplement: Supplementary file 1 [file cancers-12-02492-s001.pdf]

cancers

Article

In Situ Metabolic Characterisation of Breast Cancer and Its Potential Impact on Therapy

Gábor Petővári [1], Titanilla Dankó [1], Anna-Mária Tőkés [2], Enikő Vetlényi [1], Ildikó Krencz [1], Regina Raffay [1], Melinda Hajdu [1], Dániel Sztankovics [1], Krisztina Németh [3], Krisztina Vellai-Takács [4], András Jeney [1], Janina Kulka [2] and Anna Sebestyén [1,*]

1 1st Department of Pathology and Experimental Cancer Research, Semmelweis University, Üllői út 26, H-1085 Budapest, Hungary; gaborpetovari@gmail.com (G.P.); tita.danko@gmail.com (T.D.); eniko.vetlenyi@gmail.com (E.V.); krencz.ildiko@gmail.com (I.K.); regiraffay@gmail.com (R.R.); melindahajdu@gmail.com (M.H.); sztankovics.daniel@gmail.com (D.S.); ajeney7@gmail.com (A.J.)
2 2nd Department of Pathology, Semmelweis University, Üllői út 93, H-1091 Budapest, Hungary; tokesa1972@yahoo.co.uk (A.M.T.); janinakulka@gmail.com (J.K.)
3 MS Metabolomics Laboratory, Core Facility, Research Centre for Natural Sciences, Magyar Tudósok Blvd 2, H-1117 Budapest, Hungary; nemeth.krisztina.94@ttk.hu
4 Department of Biological Anthropology, Eötvös Loránd University, Pázmány Péter sétány 1/A, H-1117 Budapest, Hungary; takacsve@gmail.com
* Correspondence: hsebanna@gmail.com or sebestyen.anna@med.semmelweis-univ.hu

a.

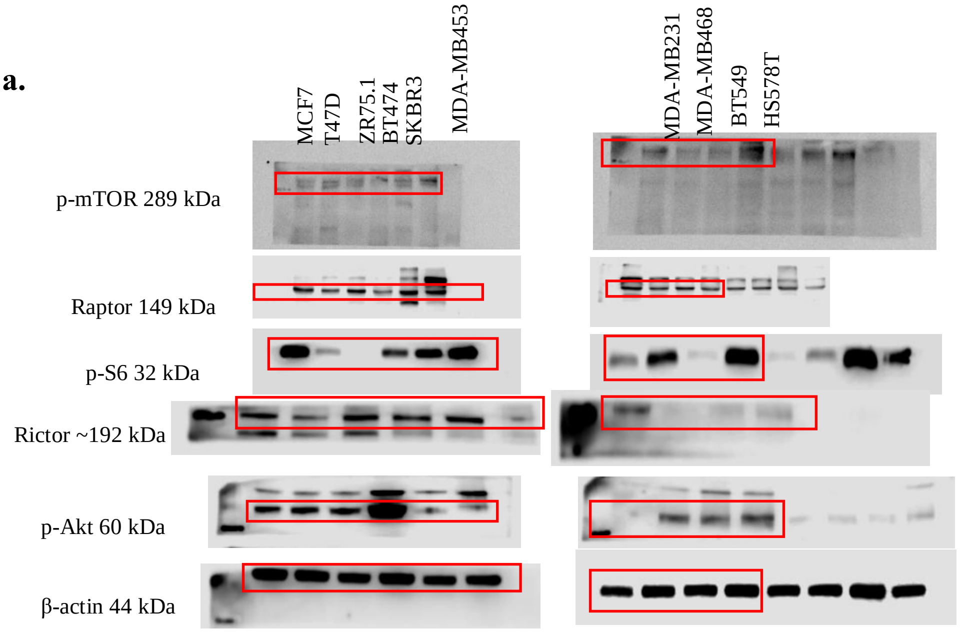

/actin	p-mTOR	Raptor	p-S6	Rictor	p-AKT
MCF7	0.009692	0.062257	0.407450	0.025115	0.037329
T47D	0.017515	0.052199	0.036214	0.013241	0.038964
ZR75.1	0.024592	0.122776	0.000000	0.051540	0.087353
BT474	0.009981	0.037310	0.103862	0.023914	0.388360
SKBR3	0.027669	0.160344	0.377664	0.048648	0.027352
MDA-MB453	0.069113	0.142969	0.719337	0.022075	0.116274
MDA-MB231	0.046778	0.173540	0.131893	0.097597	0.006706
MDA-MB468	0.010133	0.060203	0.210858	0.014854	0.197198
BT549	0.016084	0.147250	0.019389	0.027205	0.136943
HS578T	0.108190	0.034331	0.294728	0.032896	0.086534

b.

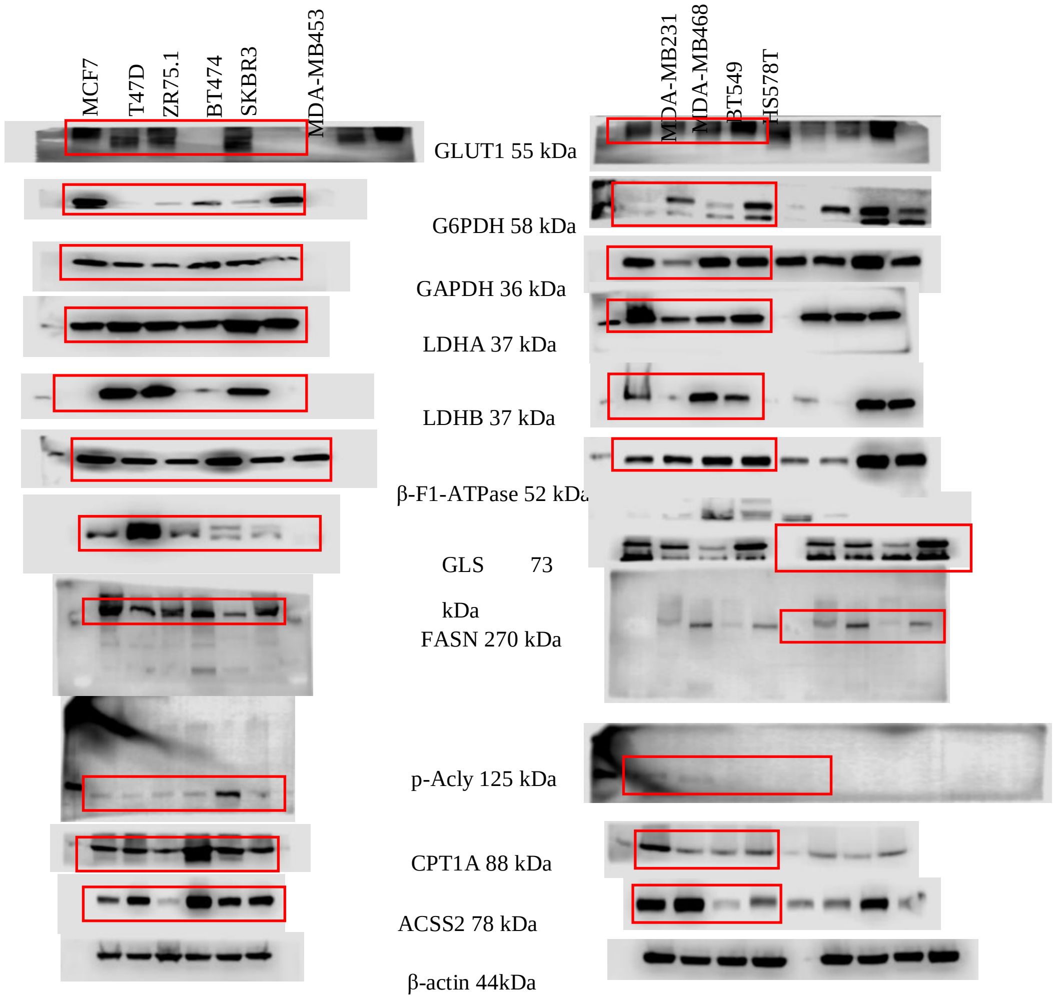

/actin	GLUT1	G6PDH	GAPDH	LDHA	LDHB	ATPB	GLS	FASN	p-ACLY	CPT1A	ACSS2
MCF7	11.511747	106.140716	140.310162	0.793090	0.002957	49.216348	0.746460	0.203983	0.004454	0.307196	0.294221
T47D	11.938963	0.619569	48.888147	1.320740	0.794520	14.507517	5.237127	0.099447	0.001154	0.369802	0.594837
ZR75.1	38.514925	13.545570	186.398882	1.869248	2.445778	57.701895	2.050475	0.075567	0.004655	0.349128	0.847643
BT474	2.932523	6.387747	61.866689	0.688476	0.199286	26.564703	0.517561	0.155221	0.004332	1.431211	3.785518
SKBR3	25.464464	2.802892	69.854358	2.307582	0.657263	8.520077	0.568121	0.031417	0.038798	1.152385	1.217293
MDA-MB453	7.238172	43.606690	29.550904	3.682152	0.012282	17.009130	0.061254	0.091424	0.022560	0.996362	2.415426
MDA-MB231	7.865481	0.021166	45.105775	2.526111	0.422794	2.687031	0.169416	0.016869	0.021432	1.727939	0.753686
MDA-MB468	17.880899	1.769838	35.981681	0.512552	0.052496	12.004453	0.272052	0.027829	0.008928	0.631166	0.723987
BT549	16.863861	0.191560	89.382108	0.572487	0.638611	7.613891	0.239257	0.002270	0.004321	0.460135	0.133667
HS578T	13.205971	0.965025	50.738034	0.758564	0.350125	5.736711	0.327101	0.010107	0.000726	0.408871	0.147433

c.

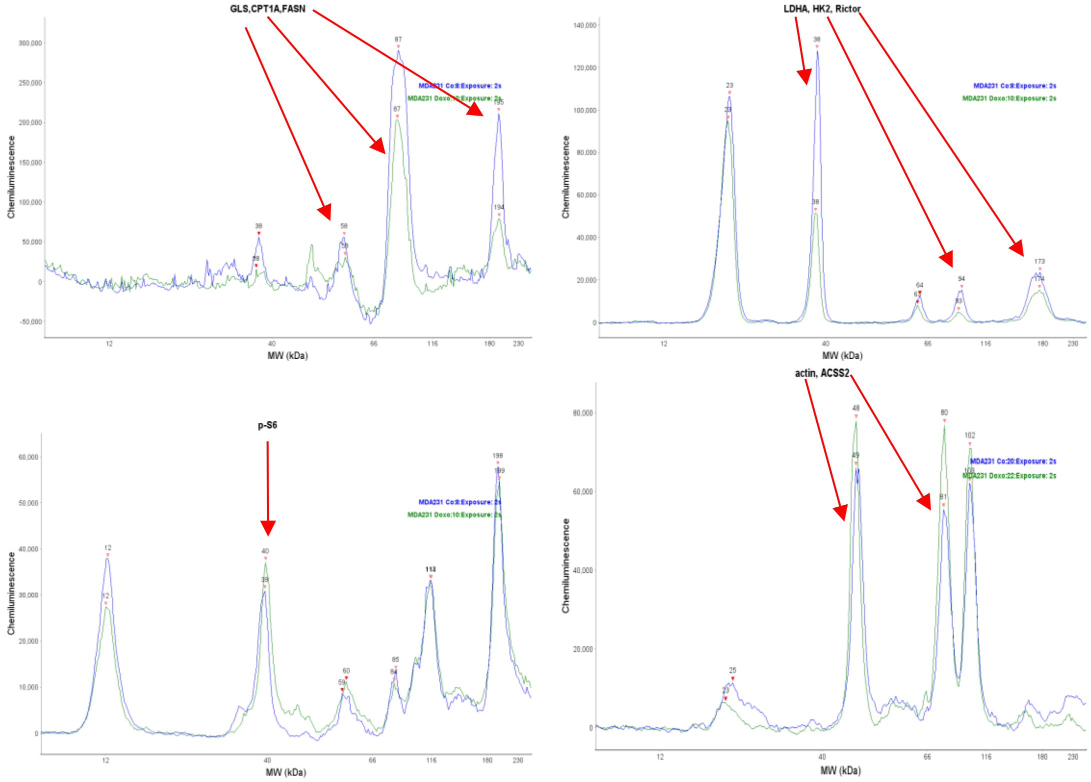

Figure S1. Additional information on Western blots and WES Simple. (a) The uncropped Western blot bands and densitometric values of the blots related to Figure 2. (b) The uncropped Western blot bands and densitometric value of the blots related to Figure 3. (c) The WES Simple electropherograms of the studied proteins related to Figure 4.

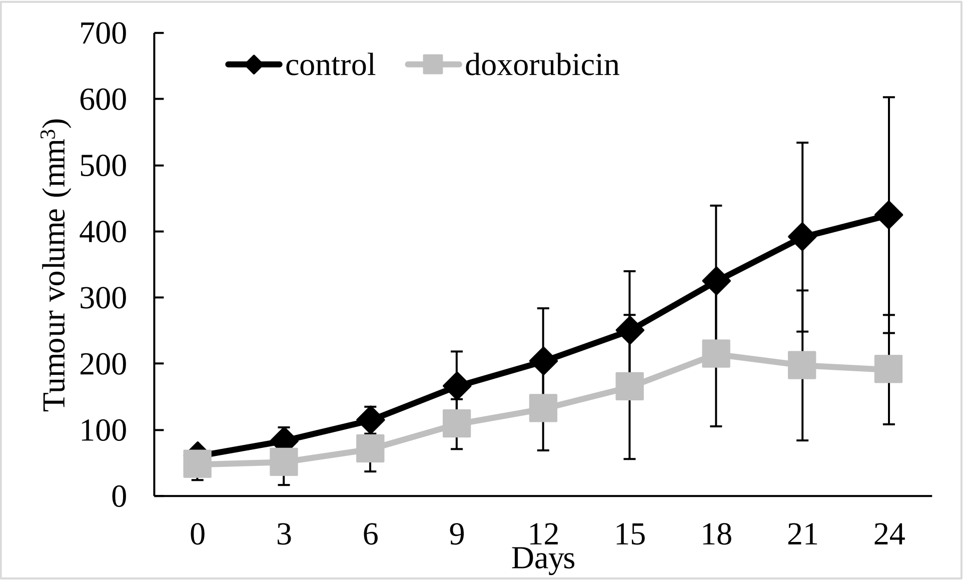

Figure S2: The growth curves of the in vivo MDA-MB231 xenografts after doxorubicin treatment. Doxorubicin treatment (2 mg/kg) reduced the growth and tumour volume (calculated by 0.52 x shorter diameter2 x longer diameter) of MDA-MB231 in vivo.

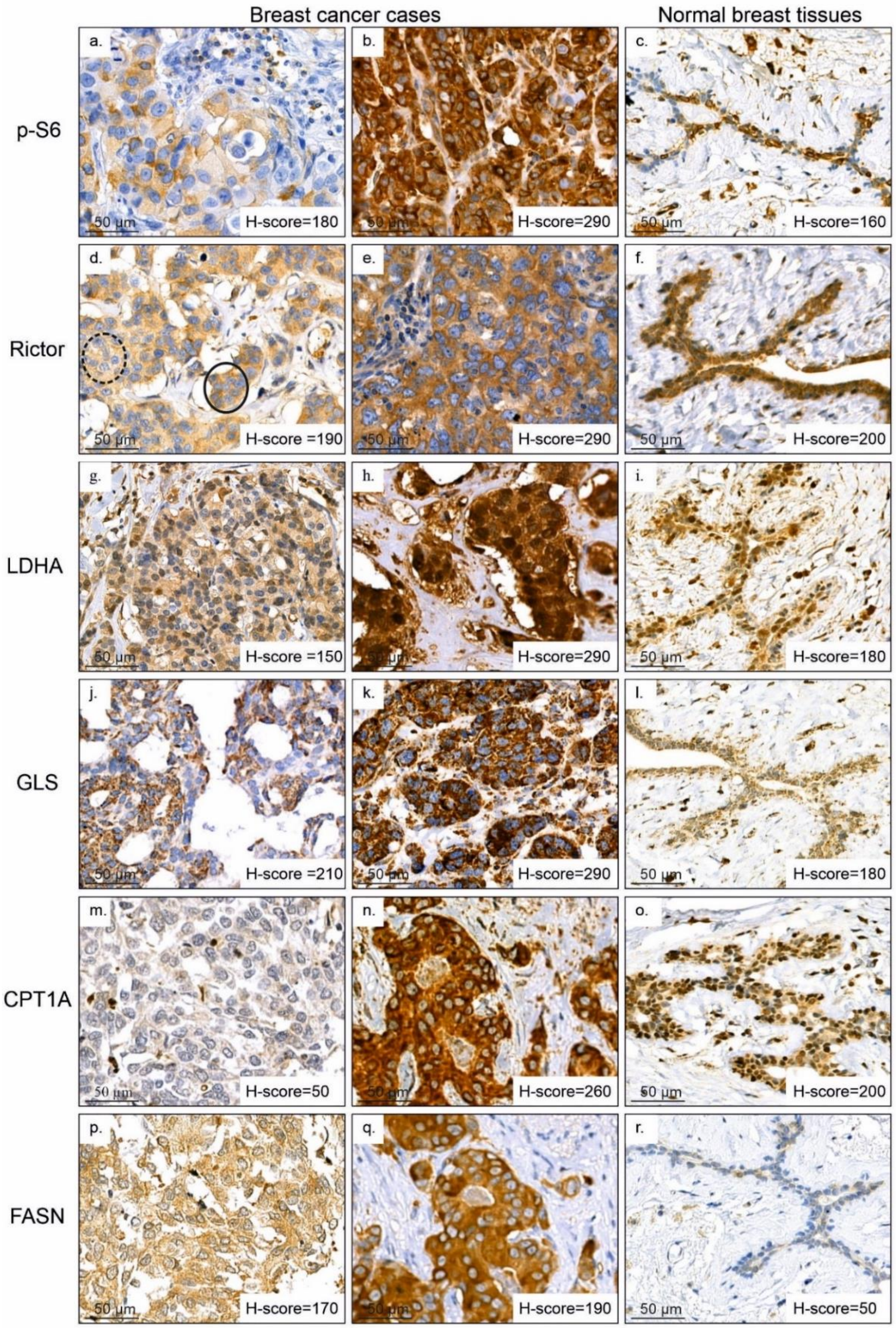

Figure S3: The expression of selected protein markers in human breast cancer tissues. IHC analysis of p-S6 (a.,b.,c.), Rictor (d., e.,f.), LDHA (g.,h.,i.), GLS (j.,k.,l.), FASN and CPT1A in formalin-fixed paraffin-embedded human tissues (breast cancer– a.,b.,d.,e.,g.,h.,j.,k.,m.,n.,p.,q. and normal tissues – c.,f.,e.,o.,r.); heterogenous stainigs and homogenous stainings were also shown in the first and second column, respectively. The intra-tumoural heterogeneity of Rictor were labelled (dotted – low staining intensity; full cirle – high staining intensity). Representative photos are shown. DAB (brown) was used as a chromogen. H-scores related to specific cases and scale bars are indicated.

Table S1. Average results from Alamar Blue and SRB tests in human breast cancer cell lines.

	**MCF7**	**T47D**	**ZR75.1**	**BT474**	**SKBR3**	**MDA-MB453**	**MDA-MB231**	**MDA-MB468**	**BT549**	**HS578T**
RAPA	73% ± 5%	57% ± 1%	70% ± 1%	70% ± 8%	56% ± 2%	48% ± 5%	78% ± 1%	80% ± 7%	80% ± 4%	55% ± 3%
PP242	40% ± 6%	24% ± 2%	47% ± 1%	54% ± 4%	30% ± 2%	50% ± 3%	45% ± 2%	80% ± 3%	55% ± 6%	44% ± 1%
GDC	70% ± 5%	24% ± 1%	**70% ± 24%**	40% ± 11%	95% ± 3%	**85% ± 17%**	86% ± 1%	95% ± 1%	80% ± 3%	64% ± 1%
3BP	10% ± 7%	10% ± 9%	25% ± 3%	45% ± 3%	85% ± 9%	30% ± 10%	30% ± 1%	10% ± 1%	30% ± 7%	55% ± 11%
BPTES	90% ± 6%	80% ± 8%	100% ± 2%	75% ± 2%	95% ± 9%	75% ± 5%	75% ± 3%	95% ± 4%	75% ± 4%	45% ± 10%
BMS	95% ± 8%	80% ± 5%	95% ± 4%	95% ± 4%	100% ± 7%	100% ± 4%	95% ± 4%	n.a.	n.a	75% ± 6%
Etomoxir	100% ± 9%	50% ± 1%	95% ± 8%	100% ± 8%	95% ± 4%	100% ± 4%	100% ± 4%	n.a.	n.a	90% ± 6%
Doxorubicin	67% ± 14%	51% ± 13%	67% ± 4%	61% ± 3%	67% ± 12%	**53% ± 21%**	76% ± 6%	75% ± 1%	**38% ± 33%**	64% ± 4%

bold: margin of error >15% (possibility of metabolic adaptation), blue: $p < 0.05$.

Table S2. KM-plotter meta-analysis of mRNA expression and clinicopathological data from breast cancer studies.

**Protein name**	**Gene Probe ID**	KM-plotter Symbol	ER+PR+/- HER2- n=1933* *except Rictor n=841		ER+PR+/-HER2+/- n=1149* *except Rictor n=407		HR- HER2+ n=251* *except Rictor n=156		TNBC n=255* *except Rictor n=161	
			Hazard Ratio	log-rank test	Hazard Ratio	log-rank test	Hazard Ratio	log-rank test	Hazard Ratio	log-rank test
**mTOR**	**202288_at**	MTOR	0.73	*0.0004***	0.73	*0.0014***	0.82	0.31	1.13	0.5745
S6	201252_at	S6	1.41	**7.8*10-5****	1.39	**0.0007****	1.24	0.2781	0.77	0.2283
Rictor	226312_at	Rictor	0.86	0.2314	0.6	*0.0011***	0.98	0.9457	0.56	*0.0401***
LDHA	200650_s_at	LDHA	1.28	**0.004****	1.45	**0.0001****	1.23	0.2857	1.4	0.1202
GLS	203159_at	GLS	0.68	*0.000014***	0.74	*0.0017***	0.87	0.4729	1.11	0.633
FASN	212218_s_at	FASN	0.95	0.5819	0.86	0.1107	1.01	0.9429	0.85	0.4485
CPT1A	203634_s_at	CPT1A	1.45	**1.9*10-5****	1.06	0.5398	0.72	0.0904	1.18	0.4363

Correlations with *better* or **worse** prognosis. * $p < 0.05$, ** $p < 0.01$.

cancers MDPI

Table S3. List of breast cancer cell lines, including their IDs, most important mutations and subtypes.

Cell line	ATCC number	Mutation	Subtype
MCF7	ATCC-HTB-22	PIK3CA, CDKN2A	Luminal A
T47D	ATCC-HTB-133	PIK3CA, TP53	
ZR75.1	ATCC-CRL-1500	PTEN	Luminal B
BT474	ATCC-HTB-20	PIK3CA, TP53	
SKBR3	ATCC-HTB-30	TP53, HER2 ampl.	HER2+
MDA-MB453	ATCC-HTB-131	PTEN, PIK3CA	
MDA-MB231	ATCC-HTB-26	TP53, CDKN2A, KRAS	TNBC
MDA-MB468	ATCC-HTB-132	PTEN, PIK3CA, TP53, RB1	
BT549	ATCC-HTB-122	PTEN, TP53, RB1	
HS578T	ATCC-HTB-126	TP53, CDKN2A, HRAS	

Table S4. List of the applied mTOR and metabolic inhibitors.

Name of inhibitor	Abbreviation	Manufacturer	Applied final concentration	Target of inhibition
rapamycin	RAPA	Sigma-Aldrich	50 ng/ml	mTORC1
PP242	PP242	Tocris Bioscience	1 µM	mTORC1/C2
BMS-303141	BMS	Sigma-Aldrich	10 µM	lipid synthesis
etomoxir	ETO	Sigma-Aldrich	50 µM	lipid oxidation
3-bromopyruvate	3BP	Sigma-Aldrich	100 µM	glycolysis
bis-2-(5-phenylacetoamido-1,3,4- thiadiazol-2-yl)-ethyl sulphide	BPTES	Sigma-Aldrich	10 µM	glutaminolysis
GDC0068	GDC	Cayman Chemical	1 µM	Akt
Doxorubicin	doxo	Sigma-Aldrich	50 ng/ml	topoisomerase II

Table S5. List of primary antibodies used for Western blot and Immunohystochemistry.

Primary antibody	Manufacturer and catalogue number	Dilutions			Target/Function/ Marker
		WB	WES	*IHC*	
phospho-mTOR (p-mTOR)	Cell Signaling (#2976)	1:1000	-	-	mTOR complex activity
Raptor	Abcam (#40768)	1:1000	-	-	mTOR complex activity
phospho-ribosomal S6 (p-S6)	Cell Signalling (#4858)	1:1000	1:50	1:100	mTOR complex activity
Rictor	Cell Signaling (#2140)	1:1000	1:50	-	mTOR complex activity
Rictor	Bethyl (A300-458A)	-	-	1:1000	mTOR complex activity
phospho-Akt (Ser473) (p-Akt)	Cell Signaling (#4060)	1:2000	-	-	mTOR complex activity
Glucose transporter 1 (GLUT1)	Abcam (#652)	1:1000	-	-	glucose transporter
Glucose-6-phosphate dehydrogenase (G6PDH)	Abcam (#133525)	1:1000	-	-	glycolysis
Glyceraldehyde 3-phosphate dehydrogenase	Abcam (#8245)	1:10000	-	-	glycolysis

(GAPDH)					
Hexokinase II (HK2)	Cell Signaling (#2867)	-	1:50	1:200	glycolysis
Lactate Dehydrogenase A (LDHA)	Cell Signaling (#3582)	1:1000	1:50	1:400	glycolysis
Lactate Dehydrogenase B (LDHB)	Abcam (#85319)	1:2000	-	-	reverse Waburg effect
Glutaminase (GLS)	Abcam (#156876)	1:1000	1:50	1:200	glutaminolysis
Fatty acid synthase (FASN)	Cell Signaling (#3180)	1:1000	1:50	1:100	fatty acid synthesis
phospho-ATP-Citrate Lyase (p-Acly)	Cell Signaling (#4331)	1:1000	-	-	fatty acid synthesis
Carnitine Palmitoyltransferase 1A (CPT1A)	Abcam (#128568)	1:1000	1:50	1:500	fatty acid oxidation
Acyl-CoA synthetase short-chain family member 2 (ACSS2)	Cell Signaling (#3658)	1:1000	1:50	1:200	acetate consumption
β-F1-ATPase (ATPB)	Abcam (#14730)	1:2000	-	-	mitochondrial oxidative phosphorylation
